# Detoxification of deoxynivalenol by pathogen-inducible tau-class glutathione transferases from wheat

**DOI:** 10.1016/j.jbc.2025.110600

**Published:** 2025-08-14

**Authors:** Herbert Michlmayr, Martin Siller, Lidija Kenjeric, Maria Doppler, Alexandra Malachova, Manuel Hofer, Christian Hametner, Wolfgang Schweiger, Barbara Steiner, Karl G. Kugler, Klaus F.X. Mayer, Hermann Buerstmayr, Rainer Schuhmacher, Rudolf Krska, Nikolaos E. Labrou, Anastassios C. Papageorgiou, Gerhard Adam

**Affiliations:** 1 Turku Bioscience Centre, University of Turku and Åbo Akademi University, Turku, Finland; 2 Department of Agricultural Sciences, Institute of Microbial Genetics (IMiG), BOKU University, Tulln, Austria; 3 FFoQSI GmbH – Austrian Competence Centre for Feed and Food Quality, Safety and Innovation, Tulln, Austria; 4 Department of Agricultural Sciences, Institute of Bioanalytics and Agro-Metabolomics, BOKU University, Tulln, Austria; 5 Core Facility Bioactive Molecules: Screening & Analysis, BOKU University, Tulln, Austria; 6 Department of Agricultural Sciences, Institute of Biotechnology in Plant Production, BOKU University, Tulln, Austria; 7 Institute of Applied Synthetic Chemistry, TU Wien, Vienna, Austria; 8 Plant Genome and Systems Biology, Helmholtz Zentrum München, Neuherberg, Germany; 9 Technical University of Munich, School of Life Sciences, Freising, Germany; 10 Institute for Global Food Security, School of Biological Sciences, Queen’s University Belfast, Northern Ireland, UK; 11 Laboratory of Enzyme Technology, Department of Biotechnology, School of Applied Biology and Biotechnology, Agricultural University of Athens, Athens, Greece

**Keywords:** deoxynivalenol, glutathione transferase, wheat, Fusarium, epoxide

## Abstract

Deoxynivalenol (DON) is a toxicologically relevant trichothecene mycotoxin frequently found in cereal products. It is a virulence factor produced by the plant pathogen *Fusarium graminearum* during cereal crop infections. Investigating plant defense mechanisms is crucial for understanding plant resistance to *F. graminearum* and identifying new biocatalysts for DON detoxification. Previous studies identified DON-thiol adducts in cereal samples, indicating partial DON detoxification by glutathione transferases (GSTs). DON possesses two electrophilic centers for thiol conjugation, resulting in either epoxide opening at C13 or Michael addition at C10. At present, information on plant GSTs that catalyze these reactions is limited. In this study, Fusarium-inducible wheat GSTs were identified by analyzing the transcriptome of Fusarium-infected wheat heads. Twelve highly induced genes of the tau and phi GST classes were heterologously expressed and purified, biochemically characterized with model substrates, and assayed for activity with DON. Use of LC-MS showed that four of the selected tau class GSTs conjugated DON to GSH by epoxide opening (DON-13-GSH) and/or the reversible Michael addition reaction (DON-10-GSH). The crystal structure of a wheat GST (herein designated “TaGST-10”) in complex with DON-13-GSH was solved at a resolution of 2.3 Å and provided insights into the binding of DON at the active site of tau class GSTs. Our results corroborate the hypothesis that enzyme-catalyzed, GSH-mediated DON detoxification may be involved in plant response to Fusarium infection.

*Fusarium* species are responsible for destructive plant diseases, such as Fusarium head blight (FHB) in small-grain cereals (wheat, rice, barley, and oats) and Gibberella stalk and ear rot in maize (1). In addition to severe losses in yield and quality, Fusarium infection leads to contamination with mycotoxins, particularly trichothecene toxins (2, 3). Trichothecenes are sesquiterpenoids with a 12,13-epoxy-trichothec-9-ene core structure (4) (Fig. 1). Their primary function is to inhibit eukaryotic protein synthesis by binding to the peptidyltransferase center of the ribosome 60S subunits (5). The epoxide on C12,13 is unusually stable and plays a key role in toxicity (6, 7). Most known trichothecene-producing fungi are plant pathogens. Trichothecenes primarily serve as virulence factors of *Fusarium* species in plants, which is well-documented in the case of deoxynivalenol (DON) produced by *Fusarium graminearum* (2) during wheat infections.

**Figure 1:**
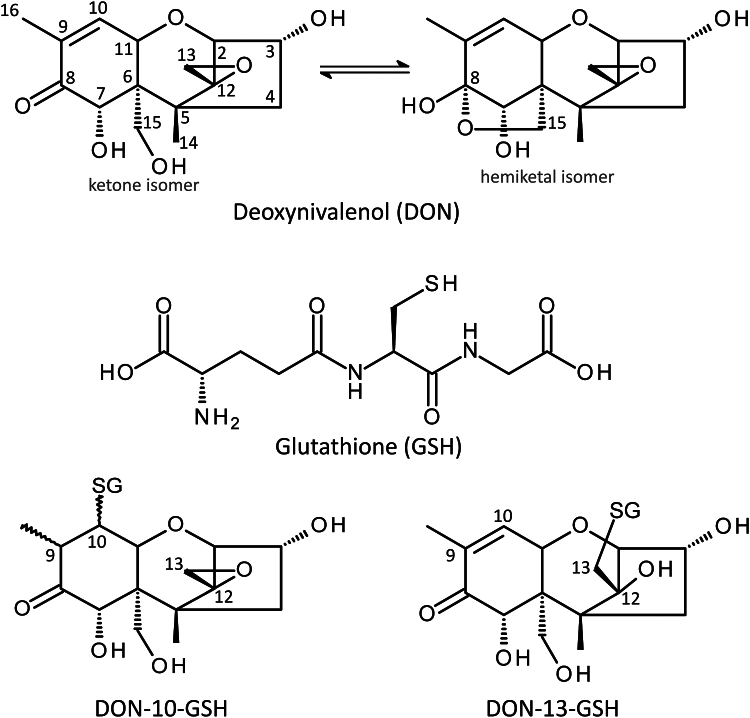
**Structures of deoxynivalenol (DON) and its glutathione (GSH) conjugates.** Michael (DON-10-GSH) and epoxide (DON-13-GSH) adducts are displayed with DON as ketone isomer (13).

DON is the most commonly detected trichothecene toxin worldwide and poses a health risk when contaminated cereals and cereal-based foods exceeding established regulatory levels are consumed (6). Investigating the enzymatic detoxification of DON (and trichothecenes in general) is important to reduce the risk to humans and livestock and to provide strategies to improve Fusarium resistance in crop plants. Apart from the epoxide group, the C3-OH group of DON is also an important factor in toxicity. Therefore, acetylation and glycosylation of C3-OH are effective detoxification mechanisms (8). However, such modifications can be reverted by glucosidases and carboxylesterases (9). Permanent detoxification by reductive de-epoxidation to de-epoxy-DON (DOM-1) is known to occur in some anaerobic ruminal/intestinal bacterial species (10, 11); however, the reaction mechanism has not yet been elucidated. Another possible route of DON detoxification is conjugation to glutathione (γ-L-glutamyl-L-cysteinyl-glycine, GSH, Fig. 1). DON presents two centers for nucleophilic attack by thiols, which can result either in the conjugation of GSH to C13 by epoxide opening or in a reversible Michael addition of GSH at C10 (Fig. 1). The respective adducts (DON-10-GSH, DON-13-GSH) resulting from the slow spontaneous reaction of DON with GSH have been previously identified and characterized (12, 13, 14).

GSH-mediated epoxide opening and Michael addition are typical reactions catalyzed by glutathione transferases (GSTs, EC 2.5.1.18) (15) and investigating the capacity of GSTs to detoxify DON appears to be promising. Especially the epoxide opening reaction is of particular interest as it implies an irreversible detoxification mechanism. GSTs comprise a multifunctional superfamily that has evolved from a thioredoxin-like ancestor. Major events in GST evolution include mutations of the ancestral catalytic cysteine to a serine and subsequently to a tyrosine (16). The ancestral cysteine GSTs mainly catalyze redox reactions and possess thioltransferase (disulfide exchange) activity (17). Serine and tyrosine GSTs mainly catalyze conjugations, and the catalytic residue promotes deprotonation of GSH resulting in nucleophilic attack of a nearby electrophilic substrate by the thiolate anion (18). The tyrosine-type GST classes alpha (GSTA), mu, and pi are animal-specific and play major roles in drug metabolism (19). The serine-type classes tau (GSTU) and phi (GSTF) are prevalent in plants and are crucial for herbicide resistance of crop plants (20). Studies on herbicide metabolism in plants have further shown that GSH conjugates rapidly undergo degradation to γ-glutamylcysteine and cysteine conjugates, which can be further processed (21, 22). Both serine- and tyrosine-type GSTs also possess GSH-dependent hydroperoxide reductase (glutathione peroxidase, GPOX) activity (23).

GST genes are abundant in plant genomes, with over 330 genes identified in wheat (24, 25), 84 in barley (26) and 91 in *Brachypodium* (27). To date, 14 evolutionary distinct GST classes have been identified in plants (18). Tau and phi class GSTs are prevalent with 200 GSTU and 87 GSTF genes reported in the wheat genome (24, 25). Several previous studies have indicated that GST genes are differentially expressed in response to Fusarium infection in plant species (28, 29, 30). However, the specific functions of such GSTs in pathogen response remain poorly understood. A phi class GST (HvGST13) from barley has been reported to be critical for Fusarium resistance by counteracting reactive oxygen species accumulation (31). A relevant question is whether plant GSTs are able to detoxify the virulence factor DON. DON-GSH conjugates have previously been identified in cereal samples, but it was not clear whether these resulted from spontaneous or enzyme-catalyzed reactions (32). A consecutive study (14) identified DON-GSH and related adducts (*e.g.*, DON-cysteine), indicating enzymatic synthesis and further processing of DON-GSH. Artificially DON-contaminated wheat spikelets (96 h after DON treatment) mainly contained Michael conjugates linked to C10 of DON, while long-term exposure to naturally contaminated wheat and oat samples (at ripening stage and after storage) primarily contained C13-epoxide adducts. While this indicates that DON may be partially detoxified by conjugation with GSH, evidence that (endogenous) plant GSTs are capable of catalysing this reaction is absent.

Therefore, the aim of this study was to identify and characterize wheat GSTs with conjugating activity toward DON. Investigation of the transcriptome of *F. graminearum*–infected wheat allowed us to identify pathogen-inducible tau class GSTs capable of catalyzing DON-10-GSH and/or DON-13-GSH formation. We determined the crystal structure of one of these GSTs (“TaGST-10”) in complex with the DON-13-GSH conjugate to obtain information on the accommodation of DON at the active site of tau-class GSTs.

## Results

### Identification of Fusarium-inducible GSTs

**Figure 2:**
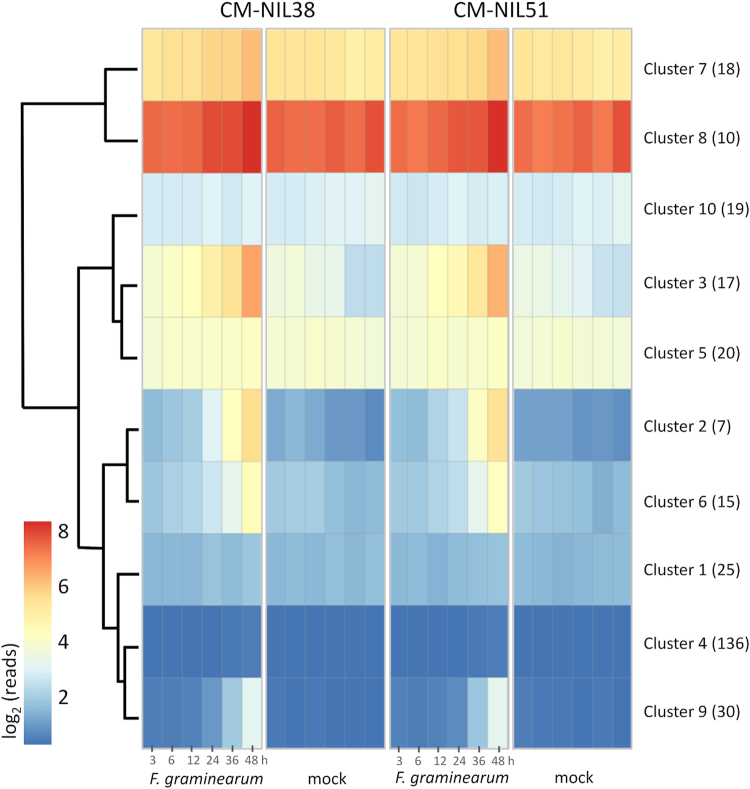
**Clustering of wheat glutathione transferase gene expresson profiles of the near isogenic lines CM-NIL38 (resistant, carrying *Fhb1* and *Qhfs.ifa-5A*) and the susceptible CM-NIL51 from log_2_-transformed RNA-Seq read counts.** Each cluster contains time-course–derived expression data (3–48 h) after inoculation with *Fusarium graminearum* or mock treatment. Clusters with no mapped reads are shown in *dark blue*, highly expressed gene clusters in *red*. The *colors* represent the cluster center (average). The number of genes in each cluster is given in *parentheses*. NIL, near-isogenic wheat line.

Data from a previously conducted RNA-Seq experiment were used to identify *Fusarium*-inducible wheat GST genes. These data were obtained from *F. graminearum*– and mock-inoculated wheat head tissues of two near-isogenic wheat lines (NILs) differing in the presence of resistant/susceptible alleles at the quantitative trait loci (QTL) *Fhb1* and *Qfhs.ifa-5A* (33). The expression profiles of 297 wheat GST genes (Table S1, annotations of the previous wheat genome assembly version, TGACv1, INSDC Assembly GCA_900067645.1, December 2015) were clustered according to their transcription patterns (Fig. 2). Clusters 2,3,6,7 and 9 contained *F. graminearum*–induced GST genes, with clusters 2 and 3 exhibiting the greatest differences in expression between the two treatments. Based on these data, 15 GSTs were initially selected as candidate genes for heterologous expression in *Escherichia coli* and further activity testing with DON (Table 1). These included all genes of cluster 2 (low basal expression, *Fusarium* induction ≥ 24 h), seven genes of cluster 3 (medium basal expression, induction ≥ 24 h), and one gene of cluster 6 (low basal expression, Fusarium induction ≥ 12–36 h). These genes were given internal designations (TaGST-01–TaGST-15), which will be used throughout the paper. The corresponding designations of the nomenclature by Wang *et al.* (24) and, if applicable, other nomenclatures found in the literature are also listed in Table 1. Three genes (TaGSTs 05, 13, and 14) were omitted during the cloning step due to unclear intron/exon annotations.

**Table 1:** Candidate wheat GST genes selected for cloning and activity testing with deoxynivalenol including nomenclature(s), and listing of expression constructs/fusion proteins (maltose-binding protein, MBP; small ubiquitin-like modifier, SUMO) used in this paper

Internal ID	Systematic name^a^	Class	Cluster	Plasmid	Fusion protein	Ensembl IDs from RNA-Seq experiment^b^	Cloned sequence (Ensembl ID, current assembly)^c^
TaGST-01	TaGSTU7	Tau	2	pSI720	N-His_6_-MBP	Traes_1AL_C40703A29	TraesCS1A02G186400.1
TaGST-02^d^	-	Tau	2	pHM96	N-His_6_-SUMO	Traes_1DL_7BCE5B151	GenBank XP_044449589.1^d^
TaGST-03	TaGSTF66	Phi	2	pSI810	N-His_6_-MBP	Traes_2DS_1063CD755	TraesCS2D02G044100.1
TaGST-04^e^	TaGSTF13^e^	Phi	2	pSI742	N-His_6_-MBP	Traes_3AS_F434A9F61	TraesCS3A02G309100.1
TaGST-05			2			Traes_4AL_396A6E2B8	
TaGST-06	TaGSTU119	Tau	2	pSI770	N-His_6_-MBP	Traes_5BL_B4E4DBF4A	TraesCS5B02G426300.1
TaGST-07	TaGSTF87	Phi	2	pSI731	N-His_6_-MBP	Traes_7DL_DEAB90162	TraesCS7D02G514500.1
TaGST-08	TaGSTU8	Tau	3	pSI718	N-His_6_-MBP	Traes_1AL_1A9EB2CBB	VAH05466.1
							TraesCS1A02G186500.1
TaGST-09	TaGSTU12	Tau	3	pSI812	N-His_6_-MBP	Traes_1AL_2103C5913	TraesCS1A02G187000.1
TaGST-10^f^	TaGSTU10	Tau	3	pSI828	N-His_6_-MBP	Traes_1AL_C64C85707	TraesCS1A02G186700.1
TaGST-11	TaGSTU11	Tau	3	pSI815	N-His_6_-MBP	Traes_1AL_CA2AFD745	TraesCS1A02G186800.1
TaGST-12	TaGSTU9	Tau	3	pHM97	N-His_6_-SUMO	Traes_1AL_CC4CF4E71	TraesCS1A02G186600.1
TaGST-13			3			Traes_1AS_00BD72553	
TaGST-14			3			Traes_7AS_B97EB9A75	
TaGST-15	TaGSTU6	Tau	6	pSI821	N-His_6_-MBP	Traes_1AL_8955C1103	TraesCS1A02G186300.1

The corresponding accession numbers of previous and current wheat genome assembly are included.

GST, glutathione transferase.

a Reference (24).

b TGACv1, INSDC Assembly GCA_900067645.1, December 2015; https://plants.ensembl.org/Triticum_aestivum/Info/Index.

c RefSeq v1.0 assembly, INSDC Assembly GCA_900519105.1, July 2018; https://plants.ensembl.org/Triticum_aestivum/Info/Index.

d Position 1D:262247455-262247781; 1D:262247865-262248248 in current assembly.

e Cloned sequence is transcript variant 1, variant 2 (TraesCS3A02G309100.2) listed as TaGSTF13 in reference (24).

f “GSTU6” in reference (47).

The remaining 12 candidates contained three phi class and nine tau class GSTs (Table 1, Fig. S1). TaGST-02 (cluster 2) is not annotated in the current wheat genome assembly version (IWGSC RefSeq v1.0, INSDC Assembly GCA_900519105.1, July 2018) and is not included in the systematic nomenclature of Wang *et al.* (24), which is based on RefSeq v1.0. Nevertheless, using the sequence of the current assembly, the gene was correctly predicted by the FGENESH web server ((34), Fig. S2).

### Conjugating activity with DON

**Figure 3:**
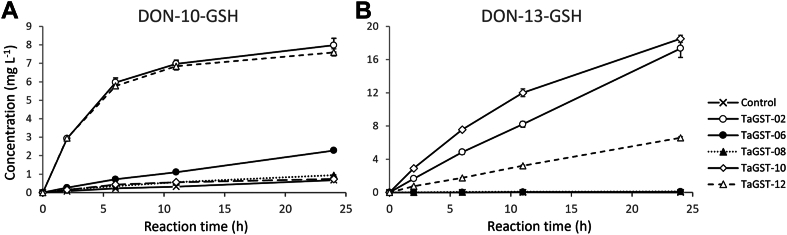
**Conjugation of deoxynivalenol by tau class glutathione transferases.** Formation of (*A*) DON-10-GSH (Michael adduct) and (*B*) DON-13-GSH (epoxide adduct) catalyzed by one-step IMAC-purified wheat GSTs. TaGST-06, TaGST-08, and TaGST-10 were expressed as *N*-His_6_-MBP-GST fusion proteins; TaGST-02 and TaGST-12 as *N*-His_6_-SUMO-GST. The assays were conducted with 5 mg ml^−1^ of the tagged GSTs and contained 30 mg l^−1^ (0.1 mM) DON, 5 mM GSH in 100 mM phosphate buffer (pH 6.5) at 20 °C. The reaction mix without added enzyme was used as negative control. DON-10-GSH and DON-13-GSH were quantified by LC-MS/MS. The values displayed represent the average of triplicate determination, error bars indicate SD. DON, deoxynivalenol; GST, glutathione transferase; MBP, maltose-binding protein.

All GSTs were expressed in a modified *E. coli* T7 Express (ΔgstA) strain to avoid a possible background from the endogenous *E. coli* GST. Activity with DON was tested with one-step IMAC-purified (*N*-His_6_-MBP or *N*-His_6_-SUMO-tagged) full-length fusion proteins (Fig. S3). Formation of DON-GSH conjugates was quantified by LC-MS/MS (Fig. 3) using DON-10-GSH and DON-13-GSH standards prepared in this study. To confirm their identity and purity, these standards were further characterized by high-resolution mass spectrometry (LC-HRMS, Fig. S4). Fragmentation of the protonated [M + H]^+^ ion with the mass/charge ratio of 604.2171 revealed significant differences in the relative abundances of fragment ions as well as differences in chromatographic retention times (RTs), most likely due to the varying polarity and steric effects introduced by GSH attachment at C10/C13 of DON. DON-13-GSH (Fig. S4*A*) exhibited higher polarity, eluting at 6.52 min, with prominent product ions at *m/z* 529.1849 (C_23_H_33_N_2_O_10_S^+^), *m/z* 499.1741 (C_22_H_31_N_2_O_9_S^+^), *m/z* 445.1638 (C_19_H_29_N_2_O_8_S^+^), *m/z* 281.0840 (C_14_H_17_O_4_S^+^), *m/z* 263.0734 (C_14_H_15_O_3_S^+^), and *m/z* 231.1014 (C_14_H_15_O_3_^+^). In contrast, DON-10-GSH (Fig. S4*B*) exhibited lower polarity and eluted at 9.66 min, with prominent product ions at *m/z* 529.1848 (C_23_H_33_N_2_O_10_S^+^), *m/z* 475.1741 (C_20_H_31_N_2_O_9_S^+^), *m/z* 162.0218 (C_5_H_8_NO_3_S^+^), *m/z* 179.0483 (C_5_H_11_N_2_O_3_S^+^), *m/z* 372.1473 (C_17_H_26_NO_6_S^+^), and *m/z* 297.1330 (C_15_H_21_O_6_^+^). Notably, emphasis should be placed on the intact DON fragment (*m/z* 297.1330), as it is present only in the DON-10-GSH spectra and absent in the DON-13-GSH spectra. These HRMS fragmentation patterns agree with the characteristic fragment patterns described by Stanic *et al.* (13).

Of the 12 candidates, TaGST-02, TaGST-06, TaGST-08, TaGST-10, and TaGST-12 displayed detectable DON-GSH adduct synthesis at 30 mg l^−1^ (0.1 mM) DON (Fig. 3). In each case, DON-10-GSH formation was significantly different from the control without enzyme in a *t* test (*p* values in Table S2), and spontaneous DON-13-GSH formation was not detected within 24 h. The time courses of adduct formation indicated that TaGST-02 and 12 catalyzed both the epoxide opening and Michael addition reactions (Fig. 3). TaGST-10 catalyzed DON-13-GSH formation but only traces of the DON-10-GSH adduct. TaGST-06 and TaGST-08 only displayed very low rates of DON-10-GSH adduct formation. With values in the pmol min^−1^ mg^−1^ range, the corresponding catalytic rates are low. Apparent specific activities with DON (sum of both adducts if applicable) inferred from the first data point (2 h reaction time) are displayed in Table 2. To confirm that adduct formation was enzyme catalyzed, we performed an independent assay (n = 5) with TaGST02 and TaGST10 (both *N*-His_6_-SUMO-tagged) additionally purified by size-exclusion chromatography and DON in molar excess (5 mM) but otherwise identical conditions. Under these conditions TaGST02 yielded approximately equal amounts of DON-10-GSH (45%) and DON-13-GSH (55%) with a total apparent specific activity of 1.1 10^−4^ ± 0.1 10^−4^ μmol min^−1^ mg^−1^. A *t* test (two-sample, two-tailed) implied significant difference to the control without enzyme with *p* = 6.0 10^−10^ for DON-10-GSH and 3.4 10^−11^ for DON-13-GSH synthesis. With TaGST10, DON-10-GSH formation was not significantly different from the control (*p* = 0.18), DON-13-GSH was synthesized with 6.3 10^−5^ ± 0.4 10^−5^ μmol min^−1^ mg^−1^ (*p* = 3.2 10^−8^).

**Table 2:** Apparent specific activities (μmol min^−1^ mg^−1^) of one-step IMAC-purified wheat glutathione transferases determined at 0.25 mM substrate concentration in 100 mM potassium phosphate (pH 6.5), 5 mM GSH at 20 °C

Candidate GST	Apparent specific activity (μmol min^−1^ mg^−1^)					
	DON	CDNB	EPNP	ETA	PEITC	CuOOH
TaGST-01	nd	nd	nd	nd	nd	nd
TaGST-02	1.17 10^−5^ ± 0.01 10^−5^	0.74 ± 0.07	nd	0.23 ± 0.03	4.9 ± 0.4	0.19 ± 0.03
TaGST-03	nd	nd	nd	nd	nd	nd
TaGST-04	nd	0.20 ± 0.01	nd	nd	0.041 ± 0.015	nd
TaGST-06	3.2 10^−7^ ± 0.03 10^−7^	1.93 ± 0.04	nd	0.21 ± 0.01	0.21 ± 0.02	<0.01
TaGST-07	nd	nd	nd	nd	0.070 ± 0.012	nd
TaGST-08	4.2 10^−8^ ± 0.5 10^−8^	0.017 ± 0.002	nd	nd	1.0 ± 0.2	nd
TaGST-09	nd	nd	nd	nd	0.46 ± 0.06	nd
TaGST-10	7.7 10^−6^ ± 0.1 10^−6^	0.46 ± 0.02	nd	0.065 ± 0.010	0.75 ± 0.11	nd
TaGST-11	nd	<0.01	nd	nd	nd	nd
TaGST-12	9.4 10^−6^ ± 0.4 10^−6^	1.1 ± 0.1	nd	0.22 ± 0.04	9.0 ± 0.5	0.25 ± 0.01
TaGST-15	nd	nd	nd	nd	0.10 ± 0.02	nd

Except for TaGST-02 and TaGST-12 (*N*-His_6_-SUMO), all GSTs were expressed as *N*-His_6_-MBP fusion proteins. The displayed values represent average ± SD of five replicates. Reads not significantly different from the control in Students *t* test (*p* > 0.05) are indicated as not detectable (nd). Activities with DON were determined in triplicate with 0.1 mM DON (Fig. 3). The results represent the sum of DON-10-GSH and DON-13-GSH synthesis rates as quantified by LC-MS.

CDNB, 1-chloro-2,4-dinitrobenzene; DON, deoxynivalenol; EPNP, 1,2-epoxy-3-(4-nitrophenoxy)propane; CuOOH, cumene hydroperoxide; PEITC, phenylethyl isothiocyanate; GST, glutathione transferase.

### Substrate specificities and kinetics

All candidate GSTs were further tested with model substrates typically used for GST characterization and representing different reaction types (Table 2). The substrates included 1-chloro-2,4-dinitrobenzene (CDNB, nucleophilic substitution), 1,2-epoxy-3-(4-nitrophenoxy) propane (EPNP, epoxide opening), phenylethyl-isothiocyanate (PEITC, nucleophilic addition), ethacrynic acid (ETA, Michael addition), and cumene hydroperoxide (CuOOH, GPOX activity). Several GSTs showed very low or no activity against these compounds. In particular, TaGST-01, TaGST-03, and TaGST-11 were completely inactive. TaGSTs 02 and 12 (94% sequence identity) showed similar activity profiles. They were active with all substrates except EPNP, which was not conjugated by any of the GSTs. TaGST-02 and TaGST-12 were also the only tested members with GPOX activity toward CuOOH. Together with TaGST-02 and 12, TaGSTs 06, 08, and 10 showed the highest conjugating activities but clearly different reaction profiles compared to TaGSTs 02 and 12. This is particularly evident by the absence of activity with ETA and CuOOH. PEITC was converted by most of the included GSTs, and particularly high-specific activities were observed with TaGST-02 and TaGST-12. Overall, these results indicated distinct reaction profiles among the tested GSTs.

Steady-state kinetic analyses were performed using TaGST-02 and TaGST-10. Except for ETA, both GSTs mainly displayed sigmoid saturation kinetics with their “hydrophobic” substrates, indicating positive cooperativity to varying extent with Hill coefficients (n) between 1.3 and 1.8 (Table 3, Figs. S5 and S6). Kinetic analysis confirmed the high activity with PEITC, with high catalytic efficiencies (*k*_cat_/*K*_0.5_) displayed by both enzymes. In particular, TaGST-02 displayed remarkably high affinity, with *K*_0.5_ in the low micromoles per liter range, accompanied by substrate inhibition with a *K*_i_ estimated at 0.14 mM PEITC. Therefore, we also tested the analog allyl isothiocyanate (AITC), which was efficiently conjugated by both GSTs but with much lower catalytic efficiencies compared to PEITC. In both cases, the lowest affinity was observed with CDNB but sufficient saturation could not be achieved within the solubility limit (approximately 2 mM in an aqueous solution). When 20% (v/v) methanol (MeOH) was added, the solubility increased to approximately 5 mM. Nevertheless, this still required extrapolation; therefore, the kinetic parameters could only be estimated with high uncertainties (Table 3).

**Table 3:** Apparent kinetic parameters of TaGST-02 and TaGST-10 with glutathione (GSH), 1-chloro-2,4-dinitrobenzene (CDNB), phenylethyl-isothiocyanate (PEITC), allyl isothiocyanate (AITC), ethacrynic acid (ETA), and cumene hydroperoxide (CuOOH)

Substrate	*K*_0.5_ or *K*_m_ (mM)	Hill coefficient (n)	Apparent maximum specific activity (μmol min^−1^ mg^−1^)	*k*_cat_ (s^−1^)	*k*_cat_/*K*_0.5_ (s^−1^ mM^−1^)
TaGST-02					
CDNB					
(5 mM GSH)	6.8 ± 5.9	1.5 ± 0.4	23 ± 17	14	2.1
GSH					
(0.5 mM CDNB)	0.25 ± 0.02	-	1.17 ± 0.03		
PEITC^a^					
(5 mM GSH)	0.0044 ± 0.0009	-	9.9 ± 0.8	6.3	1423
AITC					
(5 mM GSH)	0.10 ± 0.02	1.2 ± 0.2	5.4 ± 0.6	3.4	34
ETA					
(5 mM GSH)	0.094 ± 0.015	-	0.34 ± 0.02	0.21	2.3
CuOOH					
(5 mM GSH)	0.34 ± 0.02	1.23 ± 0.05	0.79 ± 0.02	0.50	1.5
TaGST-10					
CDNB					
(5 mM GSH)	6.5 ± 3.2	1.8 ± 0.3	42 ± 22	48	7.5
GSH					
(0.5 mM CDNB)	0.46 ± 0.01	-	0.91 ± 0.01		
PEITC					
(5 mM GSH)	0.041 ± 0.004	1.3 ± 0.1	1.7 ± 0.1	2.0	47
AITC					
(5 mM GSH)	0.080 ± 0.005	1.3 ± 0.1	0.20 ± 0.01	0.23	2.9
ETA					
(5 mM GSH)	0.023 ± 0.003	-	0.111 ± 0.003	0.13	5.5

The assays were carried out in 100 mM potassium phosphate pH 6.5, 20 °C, each measurement was performed in triplicate. The results shown are the curve fit parameters (estimate ± standard error) using either the Hill (Equation 1) or the Michaelis-Menten model (Equation 2). In case no Hill coefficient (n) is given (−), the Michaelis-Menten model was used and *K*_0.5_ equals *K*_m_. Calculation of *k*_cat_ is based on the monomeric masses of *N*-His_6_-SUMO-TaGST-02 (38.0 kDa) and *N*-His_6_-MBP-TaGST-10 (68.4 kDa).

a Haldane model of substrate inhibition (Equation 3), Ki = 0.14 ± 0.03 mM PEITC.

### Structure of TaGST-10

#### Description of the overall structure

**Figure 4:**
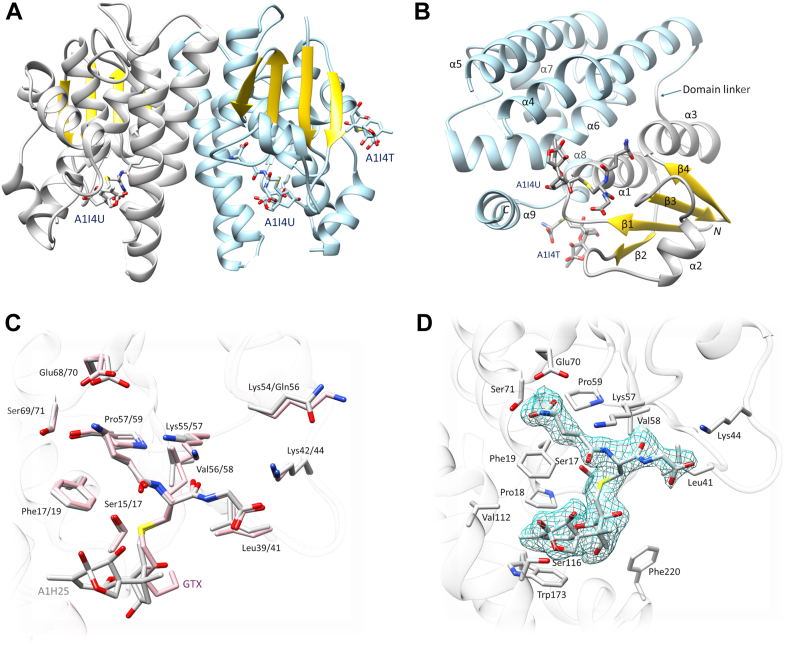
**Structure of TaGST-10with DON-13-GSH*****(PDB identifier code 9S3A).**A*, ribbon representation of the TaGST-10 dimer (chain A, *light blue*; chain B, *gray*) with DON-13-GSH (PDB identifier code A1I4U) bound at both active sites and DON-13-cysteine (PDB identifier code A1I4T) bound to chain A. *B*, monomer of TaGST-10 (chain A) with bound ligands. The *N*-terminal domain is colored *gray* (with β-strands in *gold*), the *C*-terminal domain *light blue*. The numbering of secondary structure elements follows reference (35). *C*, superposition of TaGST-10 (chain B, *gray*) on TaGSTU4 (chain B, *pink*) including their active site ligands DON-13-GSH (A1I4U) and S-hexyl-GSH (GTX), respectively. G-site residues are shown in *stick representation*, with TaGSTU4/TaGST-10 numbering, respectively. *D*, sigma-A weighed 2mFo-DFc electron density (contoured at 1.0 σ) for DON-13-GSH bound to the active site of TaGST-10 chain B. Active site residues interacting with DON-13-GSH are shown in *stick representation*. DON, deoxynivalenol; GST, glutathione transferase.

TaGST-10 was cocrystallized with its reaction product, DON-13-GSH, to observe substrate/product binding at the active site. The structure was resolved at a resolution of 2.3 Å. Data collection and refinement statistics are shown in Table 4. TaGST-10 crystallized as a dimer in the asymmetric unit and contained a molecule of DON-13-GSH in each active site. An additional ligand, modeled as DON-13-cysteine, is bound to chain A (Fig. 4). Several *N*- and *C*-terminal amino acid residues could not be traced due to missing electron density. Furthermore, the region connecting α4-α5 (residues 119–130) was difficult to trace in both chains due to unclear electron density. This was most likely caused by the high flexibility of that region. Therefore, several residues in this loop were omitted from both chains.

**Table 4:** Data collection and refinement statistics of TaGST-10

Collection statistics	
Wavelength (Å)	0.976
Space group	P 2_1_ 2 2_1_
Unit cell (Å, °)	52.4 61.9 168.8 90 90 90
Resolution range (Å)	49.9 − 2.3 (2.38 − 2.30)
Unique reflections	25,016 (2407)
Completeness (%)	99.3 (98.7)
Multiplicity	12.1 (11.4)
R_merge_	0.182 (0.978)
R_pim_	0.053 (0.287)
Mean I/sigma(I)	10.5 (3.5)
CC_1/2_	0.996 (0.935)
Wilson B-factor (Å^2^)	30.8
Refinement statistics	
Resolution range (Å)	49.9 − 2.3 (2.36 − 2.30)
Reflections used in refinement	23,653 (1696)
Reflections in free set	1280 (82)
R_work_/R_free_	0.210 (0.225)/0.258 (0.247)
CC (work)	0.932
CC (free)	0.894
Number of nonhydrogen atoms	3600
Macromolecules	3131
Ligands	152
Solvent	317
Protein residues	419
RMS (bonds) (Å)	0.012
RMS (angles) (°)	2.28
Ramachandran favored/outliers (%)	98.78/0.24
Rotamer outliers (%)	2.15
ClashScore	3.87
Mean B value (overall Å^2^)	40.59
Number of TLS groups	2
PDB identifier	9S3A

Abbreviation: TLS, translation-libration-screw.

**Figure 5:**
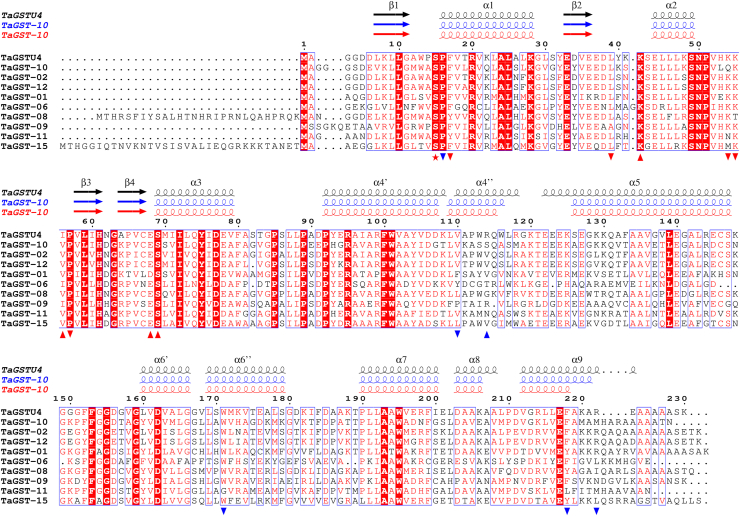
**Sequence alignment of herein investigated tau GSTs with TaGSTU4** (35)**.** Experimentally determined secondary structure elements of TaGSTU4 (chain B, 1GWC) and TaGST-10 (chain A, *blue*; chain B, *red*) are indicated. The numbering of secondary structure elements follows reference (35). The catalytic serine (Ser17) is highlighted with a *red asterisk*. G-site residues interacting with GSH are indicated by *red triangles*, H-site residues interacting with the DON moiety of DON-13-GSH in TaGST-10 by *blue triangles*. (▴) indicates polar interactions, (▾) nonpolar contact with DON-13-GSH. A corresponding phylogeny is shown in Fig. S1. Sequence alignment (ClustalW algorithm) and phylogeny (neighbour-joining tree) were created with MEGA 10 (77). The sequence alignment was annotated with ESPript 3 (78). DON, deoxynivalenol; GST, glutathione transferase.

The herbicide safener-inducible wheat tau class TaGSTU4 (35) (“TaGSTU185” according to the nomenclature in reference (24)) is the closest homolog of TaGST-10 (64% sequence identity) with a deposited structure (PDB code 1GWC). TaGST-10 displays similar overall fold and arrangement of secondary structural elements as TaGSTU4 (Fig. 5, Fig. S7*A*). Both enzymes display the classical GST fold with an *N*-terminal domain forming a thioredoxin fold (βαβ-ββα) connected by a linker (residues 82–93 in TaGST-10) to the larger, all-helical *C*-terminal domain (helices α4-α9). Superposition of TaGST-10 onto TaGSTU4 indicated high similarity in the fold of the *N*-terminal thioredoxin domain with more notable differences in the *C*-terminal domain (Fig. S7*A*). The largest differences were observed in the *N*-terminal part of helix α5 and *C*-terminal helix α9 (Fig. 5, Fig. S7*A*). Superposition of the two TaGST-10 chains is shown in Fig. S7*B*. Interface analysis (PDBePISA, Table S3) indicated that the interactions between the monomers are mainly governed by hydrophobic interactions as previously reported for TaGSTU4 (35). Polar interactions are observed between Glu80-Arg97/Arg101 and Pro67-His95 (Table S4).

#### Active site stereochemistry and binding of DON13GSH

The active site of GSTs is located at the domain interface. The GSH-binding pocket located in the *N*-terminal domain is usually termed the “G-site” and the hydrophobic substrate-binding pocket contributed by the *C*-terminal domain is termed the “H-site.” TaGST-10 residues interacting with DON-13-GSH were identified with LigPlot (Fig. S8). The corresponding residues are also highlighted in the alignment in Figure 5. Structural superposition of TaGST-10 and TaGSTU4 shows that the GSH-moieties of their bound ligands (DON-13-GSH and *S*-hexyl-GSH, respectively) assume almost identical orientations (Fig. 4*C*). Similar to *S*-hexyl-GSH in TaGSTU4, the GSH moiety of DON-13-GSH is mainly bound by polar interactions involving hydrogen bonds between Glu70 and Ser71 with the glutamyl residue, Val58 with the cysteinyl residue, and Lys44 with the glycine residue of GSH (Fig. 4, *C*, *D* and S8). The catalytic serine of TaGST-10 forms a hydrogen bond with the cysteinyl-sulfur of DON-13-GSH. Nonpolar contacts of TaGST-10 residues with the GSH moiety are formed with Phe19, Leu41, Lys57, and Pro59 (Fig. 4, *C*, *D* and S8).

In solution, the C8 ketone of DON is in equilibrium with the cyclic 8,15-hemiketal form (Fig. 1). DON (unconjugated form) primarily occurs as ketone isomer, but the hemiketal form has been reported as the favored isomer of both the DON-13-cysteine and DON-13-GSH epoxide adducts (13, 36). NMR measurements of the DON-13-GSH prepared in this study (Fig. S9) confirmed that it exists as a 92:8 mixture of the hemiketal structure and its parent ketone. The NMR spectroscopic features of the latter could not be determined due to its low abundance and heavy overlap with the hemiketal signals. A question was therefore which isomer should be modeled into the structure. The structural difference between the two isomers is relatively small (Fig. 1). During ligand modeling, we observed that both forms could, in principle, fit into the structure, and the resolution does not support this distinction. We therefore modeled DON in its hemiketal form as this is the most likely configuration according to NMR. Omit maps for the ligands are shown in Fig. S10. The DON moiety of DON-13-GSH is in close proximity to the hydrophobic residues Val12, Pro18, Trp173, and Phe220. The only polar interaction with DON is a hydrogen bond between the hydroxy group of Ser116 with the C15 oxygen of DON (Fig. 4*D*). This hydrogen bond is only present in chain B with a distance of 2.9 Å (Fig. S8). Ser116 is not conserved in tau GSTs (Fig. 5). It is replaced by a valine in both TaGST-02 and TaGST-12 and an arginine in TaGSTU4. To determine whether Ser116 of TaGST-10 has an influence on the orientation of DON and plays a role in determining synthesis rates of DON-10-GSH or DON-13-GSH by TaGST-10, we created active site mutant TaGST-10 S116V. This showed lower DON-13-GSH but increased DON-10-GSH synthesis rates than the WT TaGST-10 (Fig. S11). Similar results were observed with TaGST-10 V112S. The latter was created to test whether a more polar residue close to Ser116 would have an impact on DON conjugation. Val114 of TaGST-02 is the analogous residue of Ser116 of TaGST-10. TaGST-02 V114S showed decreased DON-10-GSH production but unaltered DON-13-GSH synthesis rates compared to the TaGST-02 WT (Fig. S11).

Chain A of TaGST-10 contained an additional ligand that was modeled as DON-13-cysteine based on the electron density (omit map in Fig. S10*C*). This may be due to partial degradation of DON-13-GSH or high flexibility resulting in lack of electron density for the rest of the molecule. DON-13-cysteine is located at the entrance of a cavity located between the two domains of chain A. This cavity is formed by residues on α1, β2, and the loop connecting α8 and α9. A polar interaction with Gln24 and nonpolar contacts with Tyr36, Ala209, Val210, Met211, and Pro212 are observed (Fig. S8, *C* and *D*).

## Discussion

Plant GSTs of the tau and phi classes are involved in responses to a wide range of biotic and abiotic external stimuli, which are generally linked to oxidative stresses (18). GSTs are an important factor in herbicide resistance in crop plants (20, 37), but their roles in endogenous plant metabolism remain insufficiently understood. It was postulated that GST reaction products are often unstable intermediates, for example, resulting from reversible reactions such as Michael additions, and therefore difficult to trace (23). Plant GSTs are known to detoxify the oxidation products of unsaturated fatty acids generated under oxidative stress (oxylipins), which typically present epoxides and Michael acceptor sites (23, 38). GSTs have also been linked to the biosynthesis of anthocyanins (39) and several sulfur-containing phytoalexins, such as glucosinolates and camalexin, where they presumably catalyze the first step to introduce sulfur (23, 40, 41, 42). Furthermore, GSTs possess ligandin noncatalytic functions using GSH as cofactor rather than as a cosubstrate (20, 23, 43, 44).

The aim of this study was to investigate whether, as first proposed by Gardiner *et al.* (29), *Fusarium*-responsive wheat GSTs are able to detoxify the mycotoxin DON, a major virulence factor of the plant pathogen *F*. *graminearum*. Considering the broad functionality of plant GSTs in stress response, as briefly summarized above, it is likely that the large number of GSTs induced during infection serves different routes in the pathogen response. DON induces oxidative stress (45) and it is likely that GSTs are involved by counteracting reactive oxygen species/reactive electrophile species generated during that process. Due to the presence of two electrophilic centers for thiol conjugation (epoxide, Michael acceptor), it appears reasonable to assume that DON is also a possible target for conjugation by plant GSTs. Therefore, we analyzed transcriptome data of *F. graminearum–*infected wheat to identify Fusarium-inducible GSTs possibly active with DON. The results indicated little difference between the two investigated wheat lines (susceptible *versus* resistant), implying that regulation of most GSTs occurs independently of the resistance QTLs *Fhb1* and *Qfhs.ifa-5A*. The largest group of genes showed very low or no induction after both treatments. This implies either involvement in different processes or a tissue-specific regulation. In *Arabidopsis*, most GSTU and GSTF genes showed the highest expression in roots (18), while the present samples were taken from wheat heads. A considerable fraction (>80) of wheat GST genes was upregulated in response to Fusarium infection in both NILs. From this group, we selected several highly induced tau and phi class GSTs for biochemical characterization and could identify four tau class members (TaGSTs 02, 06, 10, 12) with detectable activity towards DON. TaGST-02 has been previously implicated as a disease-responsive GST (Affymetrix ID TaAffx.112045.1.S1_x_at) (46). The corresponding experiment was conducted with NILs also differing in the resistance QTLs *Fhb1* and *Qfhs.ifa-5A* but in the susceptible background of the spring wheat cultivar Remus (46). TaGST-10 is identical to “TaGSTU6” previously reported to confer resistance of wheat to powdery mildew (*Blumeria graminis* f. sp. *tritici*) by interaction with a cystathionine beta-synthase domain-containing protein (47).

Activity with DON appeared considerably low compared to specific activities determined with typical GST model substrates (Table 2). Such substrates are undoubtedly useful to establish a coherent reaction profile of a catalyst in question, particularly by allowing investigation of different possible reaction types and comparison to previously characterized GSTs. However, it is difficult to assess how this reflects the natural function of a GST. For example, we observed particularly high activity with PEITC with most of the herein investigated GSTs, which was confirmed by steady state kinetics in the case of TaGST-02 and TaGST-10. Since isothiocyanates are *Brassica* metabolites, a possible physiological role of such high activity/affinity in wheat GSTs is not obvious. A similar observation was reported with poplar GSTUs (48). We further conducted assays with the substrates EPNP (epoxide) and ETA (Michael acceptor) as these represent the relevant reaction types for DON conjugation. Although activity is most likely determined by the position of the electrophile substrate relative to the deprotonated thiolate, it was previously shown that mu class GSTs are prone to catalyze epoxide opening by stabilizing transition states (49). Although a correlation of ETA conjugation with DON-10-GSH synthesis was indicated (*e.g.*, only members that conjugated ETA also synthesized DON-10-GSH), no enzyme displayed activity with the epoxide substrate EPNP, although several GSTs were able to catalyze epoxide adduct formation with DON. This discrepancy could be caused by the relatively low sensitivity of the EPNP assay. Overall, the selected GSTs displayed quite distinct activity profiles with the involved model substrates, ranging from high activity to complete inactivity. Kinetic analysis of TaGSTs 02 and 10 further indicated positive cooperativity with most substrates. This is typical for GSTs and is related to conformational changes (induced fit) upon substrate binding and intersubunit structural communication (20).

So far, GSTs have mainly been crystallized with synthetic inhibitors as ligands, and only few structures with natural substrates are available. One of the aims of this study was therefore to investigate DON-13-GSH as a case of a naturally occurring ligand, mainly to investigate how DON is oriented at the active site. The crystal structure of TaGST-10 showed that DON (*i.e.*, the DON moiety of DON-13-GSH) is mainly surrounded by hydrophobic residues. Since DON is a hydrophilic molecule (XLogP3 = −0.7, https://pubchem.ncbi.nlm.nih.gov/compound/Deoxynivalenol), this implies unfavorable binding, which is likely related to the low catalytic activities observed here. The prevalence for conjugation of DON at C10 or C13 is most likely determined by its orientation at the active site relative to the activated GSH-thiol. In case of TaGST-10, only one polar contact involving Ser116 with DON-C15-OH was observed. Of the residues that make contacts with DON, Ser116 has the lowest degree of conservation within the herein investigated tau class GSTs (Fig. 5). Replacing Ser116 by Val resulted in clearly reduced DON-13-GSH synthesis by TaGST-10 S116V. This implies that Ser116 contributes to DON binding in an orientation favoring nucleophilic attack of GSH at the C13 position of DON.

In conclusion, this study provides the first evidence that tau class wheat GSTs possess the ability to detoxify the mycotoxin DON. Although the activities with DON reported here are low, it is reasonable to speculate that GSTs with such functionality contribute at least partially to Fusarium resistance by DON detoxification. Accumulation of DON-13-GSH could result from the irreversible nature of the epoxide opening reaction and a likely redundancy of wheat GSTs with similar function. An interesting aspect in this regard is the role of the Michael addition. Due to the reversibility of the reaction, Michael adducts are difficult to trace analytically. However, as this reaction is of general importance in plant metabolism, we hypothesize that also the reversible conjugation of GSH to C10 of DON may contribute to DON resistance. For example, GSTs could stabilize the Michael conjugates until they are further processed or removed from the cytosol by GSH conjugate–specific ABC transporters (50). It is further conceivable that producers of type A trichothecenes, such as T-2, HT-2, and the recently described NX toxins (51), which lack the C-8 carbonyl, possess an advantage by evading DON detoxification through the Michael addition. These questions will be addressed in further studies. Previously, a GST that catalyzes epoxide opening of trichothecenes was reported as the causative gene of the wheat resistance QTL Fhb7 (52). It was shown that this GST, which is absent from the here investigated wheat lines, is active toward a wide range of trichothecenes and provides stable detoxification by epoxide opening (53). According to Wang *et al.* (52), the Fhb7 gene is of fungal origin (FuA class, (54), has been horizontally transferred from an endophytic *Epichloë* species to *Thinopyrum elongatum* (wheatgrass), and subsequently introgressed to wheat by distant hybridization. However, consecutive studies claimed that Fhb7 homologs are widespread among *Triticeae* and not critical for FHB resistance (55, 56). Although this case requires further clarification, it demonstrates that investigating the involvement of GST-mediated DON detoxification in Fusarium resistance of crop plants is a relevant subject. Collecting more analytical data on the presence of GSH conjugates of DON and other trichothecenes in cereal crops will provide a clearer picture on the extent of GSH detoxification of trichothecene virulence factors in plant responses.

## Experimental procedures

### Expression analysis

RNA-Seq data from the two NILs CM-NIL38 and CM-NIL51 after *F. graminearum* inoculation and mock treatment were obtained from a previous experiment (33). Both wheat lines possess the background of the highly resistant cv. CM-82036. CM-NIL38 is homozygous for the resistant alleles at the QTL *Fhb1* and *Qfhs.ifa-5A*, CM-NIL51 carries the susceptible alleles (from cv. Remus) at both QTL (33). Mapped RNA-Seq reads (fragments per kilobase million) of the 297 annotated GSTs were extracted with the Ensembl IDs of wheat genome assembly version TGACv1, INSDC Assembly GCA_900067645.1, December 2015 (https://plants.ensembl.org/Triticum_aestivum/Info/Index) The reads were clustered with the R package pheatmap (57) using the k-means algorithm (k = 10) (Table S1).

### Cloning of candidate genes and expression constructs

The candidate GST genes (Table 1) were amplified from Chinese Spring wheat DNA using a nested PCR approach. To avoid difficulties due to high sequence similarities, oligonucleotide primers were designed to target flanking regions with low similarity. Primers for the amplification of full-length genes are shown in Table S5. PCR was carried out with Q5 polymerase (New England BioLabs) or Phusion polymerase (Thermo Fisher Scientific). The PCR products were ligated to a blunt end cloning vector (pMiniT, NEB) and identity/correctness of the inserts was confirmed by Sanger sequencing. Subsequently, exons were amplified with specific primers carrying restriction sites or overlaps for recombination to fuse them to the expression vector pCA02 (58), which is a variation of pKLD116 (59), to express the GSTs with an *N*-terminal His_6_-maltose binding (MBP) fusion tag using the T7 expression system (pET21a backbone). These constructs further contain a TEV cleavage site to remove the *N*-terminal fusion tag. Exons and backbone were assembled by restriction digest/ligation, overlap-extension PCR or using the NEBuilder HiFi DNA Assembly Master Mix (NEB). Details for respective genes are given in Table S6. Additional expression constructs were created for TaGST-02, TaGST-10, and TaGST-12 genes to alternatively express them as *N*-His_6_-SUMO fusion proteins (60) in pET21a (Table S7). Active site mutants of TaGST-02 and TaGST-10 (*N*-His_6_-SUMO constructs) were created by site directed mutagenesis using primers shown in Table S8.

### Knockout of gstA in T7 Express (T7 Express ΔgstA::Kan^R^)

To eliminate background GST activity, the endogenous *E. coli* gstA gene was disrupted with the red recombination system (61). *E. coli* T7 Express (NEB New England BioLabs #C2566) carrying pKD46 was transformed with the PCR product amplified with primers Del_gstA_fw (#3818) 5′-gctatggcctgcagagcatcgg-3′ and Del_gstA_rv (#3819) 5′-ggttaaacacctggcgcgagct-3′ from the template strain JW1627-1 (62) (Yale Stock Center 9386 http://cgsc.biology.yale.edu/Strain.php?ID=107667) carrying mutation gstA785(del)::kan. Transformants were selected with kanamycin at 37 °C. Successful gene disruption was confirmed by sequencing the PCR products obtained with the primers specified above.

### Protein expression and purification

T7 Express ΔgstA::Kan^R^ was used for expression of all GSTs used in this paper. Protein production was carried out in terrific broth with 0.5 mM IPTG added at the exponential phase (A_600_ ≈ 0.5), followed by incubation at 20 °C for 20 h. Cells were harvested at 6000 *g* for 15 min, washed with PBS and resuspended in 50 mM Tris pH 7.4, 500 mM NaCl, 20 mM imidazole. The cells were sonicated on ice 3 × 1 min with intervals to cool on a Bandelin Sonopuls HD 4100 at 60% amplitude, the cell lysate was cleared at 30,000 *g*, 30 min. Protein purifications were carried out using 1 ml HisTrap crude FF columns (Cytiva) or HisPur Ni-NTA columns 3 ml (Thermo Fisher Scientific). Unbound protein was washed out with the buffer specified above and bound protein was eluted with 500 mM imidazol in the same buffer. Afterward, buffer change to 10 mM Hepes, 100 mM NaCl was carried out with Amicon Ultra-15 Centrifugal Filter Unit 10 kDa (Millipore). Protein concentrations were adjusted to about 10 mg ml^−1^. For storage at −80 °C, 10% (w/w) glycerol was added. SDS-PAGE was carried out on a 12% (w/v) polyacrylamide gel with Coomassie blue staining. Protein concentrations were determined with the Bradford protein assay using bovine serum albumin as standard. Additional size-exclusion chromatography of *N*-His_6_-SUMO-TaGST-02 and *N*-His_6_-SUMO-TaGST-10 was carried out on a Superdex 200 (HiLoad 16/600) column (Cytiva) with 50 mM phosphate buffer pH 7.0 + 150 mM NaCl at flow rate of 0.5 ml min^−1^.

### Activity assays

All enzyme assays were carried out with one-step IMAC-purified (full-length, SDS-PAGE in Fig. S3) fusion proteins in 100 mM phosphate buffer pH 6.5 at 20 °C. Assays with DON included 5 mM GSH and 30 mg l^−1^ (0.1 mM) DON. GSTs were added to 5 mg ml^−1^. Samples were taken in regular intervals by dilution with MeOH:acetic acid (9:1). The inactivated samples were stored at −20 °C and further diluted in water prior to analysis by LC-MS. Identification and quantification of DON-GSH adducts by LC-MS was carried out as described below.

Substrates for activity assays were obtained from the following sources: CDNB, Acros Organics cat. no. 160511000; EPNP, Santa Cruz Biotechnology cat. no. sc-258906; CuOOH, Fluka cat. no. 28250, Sigma-Aldrich; PEITC, Sigma-Aldrich cat.no. 253731; AITC, Sigma-Aldrich cat. no. 377430, ETA, Sigma-Aldrich cat. no. E-4754. Assays with these substrates were carried with 5 mM GSH and 0.25 mM substrate concentration in standard assays. The substrates were prepared as 20 mM stock solutions in MeOH. Peroxidase (GPOX) activity was determined with 0.25 mM CuOOH in the presence of 5 mm GSH, 0.2 mM NADPH and 0.5 IU ml^−1^ of glutathione reductase. The activity was followed by monitoring the decrease in absorbance by NADPH oxidation due to GSSG reduction. The reactions were started by enzyme (GST) addition, the assays were monitored on a Shimadzu UV-1900i spectrophotometer using the following wavelengths (nm) and extinction coefficients (mM^−1^ cm^−1^): CDNB (340, 9.6), PEITC (274, 8.89) AITC (274, 7.45), EPNP (360, 0.5), ETA (270, 5), and NADPH (340, 6.22) (63, 64). Enzyme concentrations in these assays were adjusted to obtain a linear response during measurements.

Steady-state kinetics were determined with at least seven different substrate concentrations in conditions as described above. Measurements with CDNB, GSH (0.5 mM CDNB), and CuOOH were read on a BioTek Synergy H1 hybrid reader. Due to the poor affinity with CDNB, these assays were altered to contain 20% (v/v) MeOH in order to reach CDNB concentrations up to 5 mM to achieve saturation. The results were analyzed by nonlinear model fit in R using the following equations:

(1) $$\text{Hill}\;\text{equation}\;v=\frac{V_{\max}\;{\left[S\right]}^{n}}{K_{0.5}^{n}+{\left[S\right]}^{n}}$$

(2) $$\text{Michaelis}-\text{Menten}\;\text{equation}\;v=\frac{V_{\max}\;\left[S\right]}{K_{M}+\left[S\right]}$$

(3) $$\text{Haldane}\;\;\text{model}\;\text{of}\;\text{substrate}\;\text{inhibition}\;v=\frac{V_{\max}\;\left[S\right]}{K_{M}+\left[S\right]+\frac{{\left[S\right]}^{2}}{K_{i}\;}}$$

### Synthesis and purification of DON-GSH conjugates

Liquid chromatography–mass spectrometry (LC-MS) gradient-grade acetonitrile and MeOH, as well as mass spectrometry (MS)-grade glacial acetic acid (p.a.), and formic acid 98 to 100% were purchased from Sigma-Aldrich. DON-13-GSH was synthesized enzymatically with a FuA class GST from *Trichoderma reesei* homologous to Fhb7 (unpublished data). Reaction conditions were 100 mM phosphate buffer pH 6, 1.35 mM DON, 20 mM GSH, 5 mg ml^−1^ IMAC-purified GST at 25 °C for 48 h. DON-10-GSH was synthesized by spontaneous reaction with GSH following (13). The compounds were purified by preparative HPLC on an Agilent Technologies 1100 series system using a Gemini NX C18 column (150 × 21.5 mm, 5 μm) and a guard column of the same material from Phenomenex. For DON-13-GSH purification the mobile phase consisted of aqueous acetic acid (1% v/v; solvent A) and MeOH with 1% v/v acetic acid (solvent B) at a flow rate of 17 ml min^−1^. The chromatographic gradient started with an isocratic hold at 5% of solvent B for the initial 1.5 min. At 7.5 min, a linear increase initiates, reaching 21% of solvent B. A rapid shift at 7.60 min transitions to 100% B, maintaining a wash phase until the analysis concludes at 10 min. DON-10-GSH was purified using the mobile phases consisting of aqueous formic acid (1% v/v; solvent A) and MeOH with 1% v/v formic acid (solvent B) at a flow rate of 17 ml min^−1^. The gradient started with a 3-min hold at 10% B, followed by a gradual increase to 40% B at the fifth min, in the seventh min it went to 100% B until 8.50 min for a thorough wash. Chemstation B04.03 was used for acquisition. The HPLC-diode array detector was used to monitor the individual constituents and to collect fractions. The fractions supposedly containing the analyte of interest were checked using LC-UV or LC-MS/MS. After LC-UV or LC-MS/MS analysis confirmation, extracts were pooled and the solvent was removed using a CentriVap refrigerated centrifugal concentrator (Labconco Corporation). In total 9.8 mg DON-13-GSH and 3.2 mg DON-10-GSH were obtained and used as analytical standards for consecutive LC-MS/MS measurements.

Based on HPLC-UV, DON-13-GSH represented > 95% of the total peak area (after dead volume), measured at 200 nm. No interfering signals arising from potential impurities were detected in ^1^H-NMR experiments, indicating a high purity as well. In case of DON-10-GSH, the chromophore of the DON moiety is changed (loss of the double bound and as such the conjugated system), and the UV absorbance of DON-10-GSH was extremely low. Consequently, purity assessment using HPLC-UV was not feasible for this analyte. However, the identity was confirmed using LC-HRMS. Only one major ion corresponding to DON-10-GSH was detected by HRMS (see Fig. S4), indicating the absence of detectable impurities (such as DON).

### LC–high-resolution mass spectrometry

LC-HRMS measurements were performed on an Orbitrap IQ-X Tribrid (Thermo Fisher Scientific) equipped with the heated-electrospray ionization (ESI) probe source coupled to an UHPLC-system (Vanquish-Thermo Fisher Scientific). Chromatographic separation was carried out with an Atlantis dC18 Column, 100 Å, 3 μm, 2.1 mm × 150 mm, 1/pk (Waters). The column temperature was maintained at 25 °C and flow rate was 300 μl/min, while the injection volume was 2 μl. Eluent A consisted of water and eluent B of MeOH, both containing 0.1% formic acid. The method started with elution at 3% B for 1 min, followed by a linear gradient increase to 20% B at 6.5 min and then to 100% B at 9 min. The eluent was held constant at 100% B for 1.5 min, after which it rapidly returned to 3% B at 10.6 min. The system was then re-equilibrated at 3% B for 3.4 min, resulting in a total chromatographic method runtime of 14 min. Full-scan HRMS measurements were acquired in positive mode with a scan range of *m/z* 100 to 1000 and a resolution of 120,000 (full width at half maximum at *m/z* 200). The auxiliary and sheet gas flow rates were set to 10 and 45 units. Spray voltage was set to 2950 V. For tandem mass spectrometry (MS/MS) measurements, sample-specific inclusion lists were generated and MS/MS measurements were performed in positive mode with a resolving power setting of 30,000 (full width at half maximum at *m/z* 200). Fragmentation was carried out with stepped collision energies (20, 35, 45 eV). Data were manually evaluated with the Thermo Fisher Scientific Freestyle software (https://www.thermofisher.com/order/catalog/product/OPTON-30965?SID=srch-srp-OPTON-30965).

### LC-MS/MS analysis

The analytical platform used in this study was a Triple Quad 5500+ MS/MS system (Sciex) equipped with a Turbo V ESI source coupled to a 1290 series UHPLC system (Agilent Technologies). Chromatographic separation was performed at 20 °C on a Gemini C18-column, 150 × 4.6 mm i.d., 5 μm particle size, equipped with a C18 security guard cartridge, 4 × 3 mm i.d. (both Phenomenex). Elution was carried out in binary gradient mode with a flow rate of 1000 μl min^−1^. Both mobile phases contained 5 mM ammonium acetate and were composed of MeOH/water/acetic acid (10:89:1, v/v/v; eluent A) and (97:2:1, v/v/v; eluent B), respectively. For further purification of reverse osmosis water, a Pure-lab Ultra system (ELGA Lab Water, Celle, Germany) was used. After an initial hold time of 2 min at 100% A, the proportion of B was increased linearly to 50% within 3 min. A further linear increase of B to 100% within 9 min was followed by a hold time of 4 min at 100% B, then the column was re-equilibrated at 100% A for a further 2.5 min. The injection volume was set at 5 μl. ESI-MS/MS was performed in multiple reaction monitoring (MRM) mode using fast polarity switching. The settings of the ESI source were as follows: source temperature 550 °C, curtain gas 30 psi (206.8 kPa of max. 99.5% nitrogen), ion source gas 1 (sheath gas) 80 psi (551.6 kPa of nitrogen), ion source gas 2 (drying gas) 80 psi (551.6 kPa of nitrogen), ion-spray voltage −4.5 kV, collision gas (nitrogen) medium. The column temperature was set to 20 °C. The target cycle time was 760 ms, the MS pause time was 5 ms. According to the SANTE/11312/2021 validation guidelines, two MRM transitions per analyte were acquired for accurate confirmation along with the corresponding RT. The criteria for the positive finding confirmation were as follows: (i) the ion ratio of quantifier/qualifier of the samples was within 30% of average of calibration standards from the same sequence; RTs of an analyte in the samples and in a standard solution did not differ more than by 0.1 min.

During LC-MS/MS method development, polarity-specific ionization preferences were observed, with DON-10-GSH ionizing more efficiently in negative polarity mode and DON-13-GSH in positive polarity mode. The quantifier and qualifier ions in MRM were selected through analytical standard optimization. Precursor ions were identified based on their [M + H]^+^ and [M – H]^−^ signals, and fragmentation analysis determined the most abundant and structurally relevant product ions. The quantifier ion ensured accurate quantification, whereas the qualifier ion confirmed the compound identity through consistent ion ratios. Transitions were validated by optimizing collision energy, declustering potential, and cell exit potential to enhance signal intensity and reproducibility. The final MRM method was developed to maximize specificity and sensitivity for DON-10-GSH and DON-13-GSH detection. A list of the MRM transitions for both substances is available in the supplementary material (Table S9).

### NMR spectroscopy

NMR spectra of DON-13-GSH were obtained from a solution of the sample in MeOH-d_4_ on a Bruker Avance III HD 600 FT-NMR spectrometer (operating at 600.15 MHz for ^1^H and 150.9 MHz for ^13^C; Bruker BioSpin GmbH) at ambient temperature using a Cryoprobe Prodigy probehead. Chemical shifts were established based on residual solvent resonances (3.31 ppm for ^1^H, 49.15 ppm for ^13^C). All pulse programs were obtained from the Bruker software library. The NMR data were evaluated using TopSpin 3.6 (Bruker BioSpin GmbH, https://www.bruker.com/en/products-and-solutions/mr/nmr-software/topspin.html). Structure elucidation and signal assignment were carried out based on 1D (^1^H, ^13^C-CPD) and 2D (^1^H^1^H correlation spectroscopy, ^1^H^13^C heteronuclear single quantum correlation, and ^1^H^13^C heteronuclear multiple bond correlation) NMR spectra.

### Crystallization

TaGST-10 was expressed as a *N*-His_6_-MBP fusion protein. After the IMAC purification step, the fusion tag was removed using IMAC-purified MBP-super TEV protease (65). The protein was then passed over an IMAC column again to remove the released His_6_-MBP tag and the His_6_-tagged TEV protease. The protein was concentrated to 12 mg ml^−1^ in 10 mM Hepes pH 7.0, 100 mM NaCl, 0.002% (w/v) Na azide. TaGST-10 crystals were grown with the hanging drop vapour diffusion method in 1.75 M NH_4_SO_4_, 0.1 M Na acetate (pH 4.4), and 15 mM DON-13-GSH with 10 mg mL^−1^ TaGST-10 at 17 °C. At first, no crystal growth was observed for 12 days, but crystals suitable for X-ray diffraction appeared overnight after seeding (see below) and continued to grow for two further days. The seed crystals were obtained from a previous experiment with 12-day old crystals grown in 1.75 M NH_4_SO_4_, 0.1 M Na acetate (pH 4.4), and 10 mM DON-13-GSH. The seed crystals were manually crushed using a borosilicate crystal crusher. An aliquot (0.2 μl) of the drop containing the crushed seed crystals was diluted 1:50 with the precipitation solution specified above. From this dilution, 0.2 μl were taken to seed crystallization drops (4 μl) through serial dilution by transferring 0.2 μl from a seeded drop to an unseeded drop. Before flash-cooling in liquid N_2_, the crystals selected for data collection were soaked in a cryo-protectant solution containing 1.75 M NH_4_SO_4,_ 25% (v/v) glycerol, and 8 mM DON-13-GSH.

### Structure determination and analysis

Diffraction data were collected on beamline P13 at PETRA III (DESY) at 100 K. Data were processed with XDS (66) and scaled with AIMLESS (67) from the CCP4 suite (68). The structure was solved with molecular replacement using Phaser (69) as implemented in the PHENIX suite version 1.20.1-4487-000 (70). An AlphaFold (71) model of TaGST-C10 (UniProt accession A0A3B5XZG4, AF-A0A3B5XZG4-F1-model_v4.pdb) was used as search model. The resulting solution was directed to automated model building with Autobuild as implemented in PHENIX (70). Refinement was initially carried out in an iterative process with PHENIX Refine, which included simulated annealing at 1000 K, translation-libration-screw refinement, and manual interaction in COOT (72). Ligand coordinates (DON-13-GSH, DON-13-cysteine) including restraints were generated with eLBOW and fit to the electron density using LigandFit; ligand positions/orientations were manually corrected/optimized in COOT. At the final stages, refinement was carried out using REFMAC (68, 73). Structure validation was carried out using tools in CCP4 and COOT. Omit maps for the ligands were created with the Composite Omit Map tool in PHENIX using the “simple method.”

Interactions between interfaces were analyzed with the PDBePISA webserver (https://www.ebi.ac.uk/pdbe/pisa/) (74). Protein interactions with the ligands were analyzed with LigPlot^+^ (75). Figures were created with UCSF Chimera (76) and superposition of the structures was done with the MatchMaker tool embedded in UCSF Chimera. Sequence alignment (ClustalW algorithm) and phylogeny (neighbor-joining tree) were created with MEGA 10 (77). The sequence alignment was annotated with ESPript 3 (https://espript.ibcp.fr) (78).

## Data availability

All data are included in the article or in the supporting information, the TaGST-10 structure was deposited at PDB (accession 9S3A). Raw data of RNA-Seq are available in the EBI ArrayExpress repository (http://www.ebi.ac.uk/arrayexpress/) under the accession number E-MTAB-4222.

## Supporting information

This article contains supporting information (28, 33, 35, 58, 74, 75, 77, 79).

## Conflict of interest

A patent application was filed by BOKU and Helmholtz (EP3987016A1, “Method for biotransformation of trichothecenes”) and the rights transferred to DSM Austria GmbH, now also employer of MS and WS (“dsm-firmenich”).

## References

[1] Goswami R.S., Kistler H.C. (2004). Heading for disaster: fusarium graminearum on cereal crops. Mol. Plant Pathol..

[2] Proctor R.H., McCormick S.P., Kim H.S., Cardoza R.E., Stanley A.M., Lindo L. (2018). Evolution of structural diversity of trichothecenes, a family of toxins produced by plant pathogenic and entomopathogenic fungi. Plos Pathog..

[3] Chen Y., Kistler H.C., Ma Z. (2019). Trichothecene mycotoxins: biosynthesis, regulation, and management. Annu. Rev. Phytopathol.

[4] McCormick S.P., Stanley A.M., Stover N.A., Alexander N.J. (2011). Trichothecenes: from simple to complex mycotoxins. Toxins.

[5] de Loubresse N.G., Prokhorova I., Holtkamp W., Rodnina M.V., Yusupova G., Yusupov M. (2014). Structural basis for the inhibition of the eukaryotic ribosome. Nature.

[6] Foroud N.A., Baines D., Gagkaeva T.Y., Thakor N., Badea A., Steiner B. (2019). Trichothecenes in cereal grains - an update. Toxins (Basel).

[7] Wang W., Zhu Y., Abraham N., Li X.-Z., Kimber M., Zhou T. (2021). The ribosome-binding mode of trichothecene mycotoxins rationalizes their structure-activity relationships. Int. J. Mol. Sci..

[8] Karlovsky P. (2011). Biological detoxification of the mycotoxin deoxynivalenol and its use in genetically engineered crops and feed additives. Appl. Microbiol. Biotechnol..

[9] Berthiller F., Crews C., Dall’Asta C., Saeger S.D., Haesaert G., Karlovsky P. (2013). Masked mycotoxins: a review. Mol. Nutr. Food Res..

[10] Binder E.-M., Binder J. (2004).

[11] Springler A., Hessenberger S., Reisinger N., Kern C., Nagl V., Schatzmayr G. (2017). Deoxynivalenol and its metabolite deepoxy-deoxynivalenol: multi-parameter analysis for the evaluation of cytotoxicity and cellular effects. Mycotoxin Res..

[12] Stanic A., Uhlig S., Solhaug A., Rise F., Wilkins A.L., Miles C.O. (2015). Nucleophilic addition of thiols to deoxynivalenol. J. Agric. Food Chem..

[13] Stanic A., Uhlig S., Sandvik M., Rise F., Wilkins A.L., Miles C.O. (2016). Characterization of deoxynivalenol–glutathione conjugates using nuclear magnetic resonance spectroscopy and liquid chromatography–high-resolution mass spectrometry. J. Agric. Food Chem..

[14] Uhlig S., Stanic A., Hofgaard I.S., Kluger B., Schuhmacher R., Miles C.O. (2016). Glutathione-conjugates of deoxynivalenol in naturally contaminated grain are primarily linked via the epoxide group. Toxins (Basel).

[15] Salinas A.E., Wong M.G. (1999). Glutathione S-transferases-a review. Curr. Med. Chem..

[16] Frova C. (2006). Glutathione transferases in the genomics era: new insights and perspectives. Biomol. Eng..

[17] Lallement P.-A., Brouwer B., Keech O., Hecker A., Rouhier N. (2014). The still mysterious roles of cysteine-containing glutathione transferases in plants. Front. Pharmacol..

[18] Sylvestre-Gonon E., Law S.R., Schwartz M., Robe K., Keech O., Didierjean C. (2019). Functional, structural and biochemical features of plant Serinyl-Glutathione transferases. Front. Plant Sci..

[19] Hayes J.D., Flanagan J.U., Jowsey I.R. (2005). Glutathione transferases. Annu. Rev. Pharmacol. Toxicol..

[20] Labrou N.E., Papageorgiou A.C., Pavli O., Flemetakis E. (2015). Plant GSTome: structure and functional role in xenome network and plant stress response. COBIOT.

[21] Brazier-Hicks M., Evans K.M., Cunningham O.D., Hodgson D.R.W., Steel P.G., Edwards R. (2008). Catabolism of glutathione conjugates in Arabidopsis thaliana. Role in metabolic reactivation of the herbicide safener fenclorim. J. Biol. Chem..

[22] Cummins I., Dixon D.P., Freitag-Pohl S., Skipsey M., Edwards R. (2011). Multiple roles for plant glutathione transferases in xenobiotic detoxification. Drug Metab. Rev..

[23] Dixon D.P., Skipsey M., Edwards R. (2010). Roles for glutathione transferases in plant secondary metabolism. Phytochemistry.

[24] Wang R., Ma J., Zhang Q., Wu C., Zhao H., Wu Y. (2019). Genome-wide identification and expression profiling of glutathione transferase gene family under multiple stresses and hormone treatments in wheat (Triticum aestivum L.). BMC Genom..

[25] Hao Y., Xu S., Lyu Z., Wang H., Kong L., Sun S. (2021). Comparative analysis of the glutathione S-Transferase gene family of four *triticeae* species and transcriptome analysis of GST genes in common wheat responding to salt stress. Int. J. Genomics.

[26] Rezaei M.K., Shobbar Z.S., Shahbazi M., Abedini R., Zare S. (2013). Glutathione S-transferase (GST) family in barley: identification of members, enzyme activity, and gene expression pattern. J. Plant Physiol..

[27] Gallé Á., Benyó D., Gallé Á., Benyó D., Csiszár J., Györgyey J. (2019). Genome-wide identification of the glutathione transferase superfamily in the model organism Brachypodium distachyon. Funct. Plant Biol..

[28] Zhou W., Kolb F.L., Riechers D.E. (2005). Identification of proteins induced or upregulated by Fusarium head blight infection in the spikes of hexaploid wheat (Triticum aestivum). Genome.

[29] Gardiner S.A., Boddu J., Berthiller F., Hametner C., Stupar R.M., Adam G. (2010). Transcriptome analysis of the barley–deoxynivalenol interaction: evidence for a role of glutathione in deoxynivalenol detoxification. Mol. Plant Microbe Interact..

[30] Pasquet J.C., Chaouch S., Macadre C., Balzergue S., Huguet S., Martin-Magniette M.L. (2014). Differential gene expression and metabolomic analyses of Brachypodium distachyon infected by deoxynivalenol producing and non-producing strains of Fusarium graminearum. BMC Genom..

[31] Wahibah N.N., Tsutsui T., Tamaoki D., Sato K., Nishiuchi T. (2018). Expression of barley Glutathione S-Transferase13 gene reduces accumulation of reactive oxygen species by trichothecenes and paraquat in Arabidopsis plants. Plant Biotechnol. (Tokyo).

[32] Kluger B., Bueschl C., Lemmens M., Berthiller F., Häubl G., Jaunecker G. (2013). Stable isotopic labelling-assisted untargeted metabolic profiling reveals novel conjugates of the mycotoxin deoxynivalenol in wheat. Anal. Bioanal. Chem..

[33] Schweiger W., Steiner B., Vautrin S., Nussbaumer T., Siegwart G., Zamini M. (2016). Suppressed recombination and unique candidate genes in the divergent haplotype encoding Fhb1, a major Fusarium head blight resistance locus in wheat. Theor. Appl. Genet..

[34] Salamov A.A., Solovyev V.V. (2000). Ab initio gene finding in drosophila genomic DNA. Genome Res..

[35] Thom R., Cummins I., Dixon D.P., Edwards R., Cole D.J., Lapthorn A.J. (2002). Structure of a tau class glutathione S-transferase from wheat active in herbicide detoxification. Biochemistry.

[36] Stanic A., Uhlig S., Solhaug A., Rise F., Wilkins A.L., Miles C.O. (2016). Preparation and characterization of cysteine adducts of deoxynivalenol. J. Agric. Food Chem..

[37] Riechers D.E., Kreuz K., Zhang Q. (2010). Detoxification without intoxication: herbicide safeners activate plant defense gene expression. Plant Physiol..

[38] Dixon D.P., Edwards R. (2018). Protein-Ligand fishing in planta for biologically active natural products using glutathione transferases. Front. Plant Sci..

[39] Eichenberger M., Schwander T., Hüppi S., Kreuzer J., Mittl P.R.E., Peccati F. (2023). The catalytic role of glutathione transferases in heterologous anthocyanin biosynthesis. Nat. Catal..

[40] Parisy V., Poinssot B., Owsianowski L., Buchala A., Glazebrook J., Mauch F. (2007). Identification of PAD2 as a γ-glutamylcysteine synthetase highlights the importance of glutathione in disease resistance of Arabidopsis. Plant J..

[41] Geu-Flores F., Møldrup M.E., Böttcher C., Olsen C.E., Scheel D., Halkier B.A. (2011). Cytosolic γ-glutamyl peptidases process glutathione conjugates in the biosynthesis of glucosinolates and camalexin in Arabidopsis. Plant Cell.

[42] Piślewska-Bednarek M., Nakano R.T., Hiruma K., Pastorczyk M., Sanchez-Vallet A., Singkaravanit-Ogawa S. (2018). Glutathione transferase U13 functions in pathogen-triggered glucosinolate metabolism. Plant Physiol..

[43] Licciardello C., D’Agostino N., Traini A., Recupero G.R., Frusciante L., Chiusano M.L. (2014). Characterization of the glutathione S-transferase gene family through ESTs and expression analyses within common and pigmented cultivars of citrus sinensis (L.) osbeck. BMC Plant Biol..

[44] Nianiou-Obeidat I., Madesis P., Kissoudis C., Voulgari G., Chronopoulou E., Tsaftaris A. (2017). Plant glutathione transferase-mediated stress tolerance: functions and biotechnological applications. Plant Cell Rep..

[45] Mishra S., Dwivedi P.D., Pandey H.P., Das M. (2014). Role of oxidative stress in deoxynivalenol induced toxicity. Food Chem. Toxicol..

[46] Schweiger W., Steiner B., Ametz C., Siegwart G., Wiesenberger G., Berthiller F. (2013). Transcriptomic characterization of two major F usarium resistance quantitative trait loci (QTL s), F hb1 and Q fhs. ifa-5A, identifies novel candidate genes. Mol. Plant Pathol..

[47] Wang Q., Guo J., Jin P., Guo M., Guo J., Cheng P. (2022). Glutathione S-transferase interactions enhance wheat resistance to powdery mildew but not wheat stripe rust. Plant Physiol..

[48] Musdal Y., Mannervik B. (2015). Substrate specificities of two tau class glutathione transferases inducible by 2,4,6-trinitrotoluene in poplar. Biochim. Biophys. Acta Gen..

[49] Armstrong R., Sir Barton D., Nakanishi K., Meth-Cohn O. (1999).

[50] Lu Y.P., Li Z.S., Rea P.A. (1997). AtMRP1 gene of Arabidopsis encodes a glutathione S-conjugate pump: isolation and functional definition of a plant ATP-binding cassette transporter gene. Proc. Natl. Acad. Sci. U. S. A.

[51] Varga E., Wiesenberger G., Hametner C., Ward T.J., Dong Y., Schöfbeck D. (2015). New tricks of an old enemy: isolates of Fusarium graminearum produce a type A trichothecene mycotoxin. Environ. Microbiol..

[52] Wang H., Sun S., Ge W., Zhao L., Hou B., Wang K. (2020). Horizontal gene transfer of Fhb7 from fungus underlies Fusarium head blight resistance in wheat. Science.

[53] Hou B., Wang D., Yan F., Cheng X., Xu Y., Xi X. (2024). Fhb7-GST catalyzed glutathionylation effectively detoxifies the trichothecene family. Food Chem..

[54] Mathieu Y., Prosper P., Buée M., Dumarçay S., Favier F., Gelhaye E. (2012). Characterization of a Phanerochaete chrysosporium glutathione transferase reveals a novel structural and functional class with ligandin properties. J. Biol. Chem..

[55] Guo X., Wang M., Kang H., Zhou Y., Han F. (2022). Distribution, polymorphism and function characteristics of the GST-Encoding Fhb7 in triticeae. Plants.

[56] Guo X., Shi Q., Wang M., Yuan J., Zhang J., Wang J. (2023). Functional analysis of the glutathione S-transferases from Thinopyrum and its derivatives on wheat Fusarium head blight resistance. Plant Biotechnol. J..

[57] Kolde R. (2012). Pheatmap: pretty heatmaps. R. Package Version.

[58] Michlmayr H., Varga E., Lupi F., Malachová A., Hametner C., Berthiller F. (2017). Synthesis of mono- and di-glucosides of zearalenone and α-/β-zearalenol by recombinant barley glucosyltransferase HvUGT14077. Toxins.

[59] Rocco C.J., Dennison K.L., Klenchin V.A., Rayment I., Escalante-Semerena J.C. (2008). Construction and use of new cloning vectors for the rapid isolation of recombinant proteins from Escherichia coli. Plasmid.

[60] Kuo D., Nie M., Courey A.J. (2014). SUMO as a solubility tag and in vivo cleavage of SUMO fusion proteins with Ulp1. Methods Mol. Biol..

[61] Datsenko K.A., Wanner B.L. (2000). One-step inactivation of chromosomal genes in Escherichia coli K-12 using PCR products. Proc. Natl. Acad. Sci. U. S. A.

[62] Baba T., Ara T., Hasegawa M., Takai Y., Okumura Y., Baba M. (2006). Construction of Escherichia coli K-12 in-frame, single-gene knockout mutants: the Keio collection. Mol. Syst. Biol..

[63] Habig W.H., Pabst M.J., Jakoby W.B. (1974). Glutathione S-transferases. The first enzymatic step in mercapturic acid formation. J. Biol. Chem..

[64] Kolm R.H., Danielson U.H., Zhang Y., Talalay P., Mannervik B. (1995). Isothiocyanates as substrates for human glutathione transferases: structure-activity studies. Biochem. J..

[65] Keeble A.H., Yadav V.K., Ferla M.P., Bauer C.C., Chuntharpursat-Bon E., Huang J. (2022). DogCatcher allows loop-friendly protein-protein ligation. Cell Chem. Biol.

[66] Kabsch W. (2010). Integration, scaling, space-group assignment and post-refinement. Acta Crystallogr. D Biol. Crystallogr..

[67] Evans P.R., Murshudov G.N. (2013). How good are my data and what is the resolution?. Acta Crystallogr. D Biol. Crystallogr..

[68] Agirre J., Atanasova M., Bagdonas H., Ballard C.B., Baslé A., Beilsten-Edmands J. (2023). The CCP4 suite: integrative software for macromolecular crystallography. Acta Crystallogr. D Biol. Crystallogr..

[69] McCoy A.J., Grosse-Kunstleve R.W., Adams P.D., Winn M.D., Storoni L.C., Read R.J. (2007). Phaser crystallographic software. J. Appl. Cryst..

[70] Adams P.D., Afonine P.V., Bunkóczi G., Chen V.B., Davis I.W., Echols N. (2010). PHENIX: a comprehensive Python-based system for macromolecular structure solution. Acta Crystallogr. D Biol. Crystallogr..

[71] Jumper J., Evans R., Pritzel A., Green T., Figurnov M., Ronneberger O. (2021). Highly accurate protein structure prediction with AlphaFold. Nature.

[72] Emsley P., Lohkamp B., Scott W.G., Cowtan K. (2010). Features and development of Coot. Acta Crystallogr. D Biol. Crystallogr..

[73] Murshudov G.N., Vagin A.A., Dodson E.J. (1997). Refinement of macromolecular structures by the maximum-likelihood method. Acta Crystallogr. D Biol. Crystallogr..

[74] Krissinel E., Henrick K. (2007). Inference of macromolecular assemblies from crystalline state. J. Mol. Biol..

[75] Laskowski R.A., Swindells M.B. (2011). LigPlot+: multiple ligand-protein interaction diagrams for drug discovery. J. Chem. Inf. Model.

[76] Pettersen E.F., Goddard T.D., Huang C.C., Couch G.S., Greenblatt D.M., Meng E.C. (2004). UCSF Chimera--a visualization system for exploratory research and analysis. J. Comput. Chem..

[77] Tamura K., Stecher G., Kumar S. (2021). MEGA11: molecular evolutionary genetics analysis version 11. Mol. Biol. Evol..

[78] Robert X., Gouet P. (2014). Deciphering key features in protein structures with the new ENDscript server. Nucleic Acids Res..

[79] Cummins I., O’Hagan D., Jablonkai I., Cole D.J., Hehn A., Werck-Reichhart D. (2003). Cloning, characterization and regulation of a family of phi class glutathione transferases from wheat. Plant Mol. Biol..

