# Supplementary material for: Detoxification of deoxynivalenol by pathogen-inducible tau-class glutathione transferases from wheat
Source: J Biol Chem. 2025 Aug 14;301(10):110600. doi: 10.1016/j.jbc.2025.110600 (PMC12624782; doi:10.1016/j.jbc.2025.110600)
Supplement: Supporting Figures and Tables [file mmc1.pdf]

Detoxification of deoxynivalenol by pathogen-inducible tau-class glutathione transferases from wheat

Supporting Information

Herbert Michlmayr[1,2], Martin Siller[2,$], Lidija Kenjeric[3,4], Maria Doppler[4,5], Alexandra Malachova[3,4], Manuel Hofer[6, †], Christian Hametner[7], Wolfgang Schweiger[6,$], Barbara Steiner[6], Karl G. Kugler[8,], Klaus F. X. Mayer[8,9], Hermann Buerstmayr[6], Rainer Schuhmacher[4], Rudolf Krska[4,10], Nikolaos E. Labrou[11], Anastassios C. Papageorgiou[1*], Gerhard Adam[2]

[1] Turku Bioscience Centre, University of Turku and Åbo Akademi University, Tykistökatu 6, Turku 20520, Finland

[2] BOKU University; Department of Agricultural Sciences, Institute of Microbial Genetics (IMiG), Konrad-Lorenz-Strasse 24, 3430 Tulln, Austria

[3] FFoQSI GmbH – Austrian Competence Centre for Feed and Food Quality, Safety and Innovation, Technopark 1C, 3430 Tulln, Austria

[4] BOKU University, Department of Agricultural Sciences, Institute of Bioanalytics and Agro-Metabolomics, Konrad-Lorenz-Strasse 20, 3430 Tulln, Austria

[5] BOKU University, Core Facility Bioactive Molecules: Screening & Analysis, Konrad-Lorenz-Strasse 20, 3430 Tulln, Austria

[6] BOKU University, Department of Agricultural Sciences, Institute of Biotechnology in Plant Production, Konrad-Lorenz-Strasse 20, 3430 Tulln, Austria

[7]Institute of Applied Synthetic Chemistry, Vienna University of Technology, Vienna, Austria

[8] Plant Genome and Systems Biology, Helmholtz Zentrum München, Neuherberg, Germany

[9]Technical University of Munich, School of Life Sciences, Freising, Germany

[10] Institute for Global Food Security, School of Biological Sciences, Queen's University Belfast, 19 Chlorine Gardens Belfast BT9 5DL, Northern Ireland

[11] Laboratory of Enzyme Technology, Department of Biotechnology, School of Applied Biology and Biotechnology, Agricultural University of Athens, 75 Iera Odos Street, GR-118 55 Athens, Greece

Current addresses

[$] dsm-firmenich ANH Research Center Tulln, TFZ Tulln, Technopark 1, 3430 Tulln, Austria

[†] Institute of Medical Genetics, Center for Pathobiochemistry and Genetics, Medical University of Vienna, 1090 Vienna, Austria

Content

Table S1 (Excel sheet, uploaded as separate file). Expression of wheat glutathione transferase (GST) genes in the near isogenic lines CM-NIL38 (resistant, carrying *Fhb1* and *Qhfs.ifa-5A*) and the susceptible CM-NIL51. Displayed are mapped RNA-seq read counts (Fragments Per Kilobase Million, FPKM) of time-course-derived expression data (3–48 h) after inoculation with *Fusarium graminearum* or mock treatment. Clusters obtained from $\log_2$-transformed read counts are indicated in the last column. The gene IDs are the Ensembl IDs of wheat genome assembly version TGACv1, INSDC Assembly GCA_900067645.1, Dec 2015 (https://plants.ensembl.org/Triticum_aestivum/Info/Index). Raw data of RNA sequencing are available in the EBI ArrayExpress (http://www.ebi.ac.uk/arrayexpress/) repository under the accession number E-MTAB-4222 (33).

Table S2. *p* values of t-test (two-sided) of DON-10-GSH synthesis related to Fig. 3

Table S3. Interface analysis summary of the TaGST-10 dimer (PDB code 9S3A) with PDBePISA.

Table S4. Hydrogen bonds and salt bridges involved in dimerization of TaGST-10.

Table S5. Oligonucleotide primers for amplification of wheat GST genes from genomic wheat DNA (cv. Chinese Spring).

Table S6. Oligonucleotide primers for amplification of wheat GST gene exons, assembly and cloning into expression vector pCA02.

Table S7. Oligonucleotide primers for creating TaGST-02, TaGST-10 and TaGST-12 constructs for expression as *N*-His_6-SUMO-tagged proteins in pET21a.

Table S8. Oligonucleotide primers for site directed mutagenesis of TaGST02 and TaGST10.

Table S9. Multiple reaction monitoring transitions of DON-10-GSH and DON-13-GSH.

Figure S1. Neighbor-joining tree of wheat GSTs used in this study.

Figure S2. FGENESH (http://www.softberry.com/) prediction of the TaGST-02 gene.

Figure S3. SDS-PAGE of one-step IMAC purified wheat GSTs

Figure S4. LC-HRMS analysis of DON-13-GSH and DON-10-GSH

Figure S5. Steady state kinetic analysis of TaGST-02

Figure S6. Steady state kinetic analysis of TaGST-10

Figure S7. (**A**) Superposition of TaGST-10 (9S3A) on TaGSTU4 (1GWC). (**B**) Superposition of TaGST-10 chain A with chain B.

Figure S8. LigPlot representations of TaGST-10 residues interacting with ligands bound to chain A.

Figure S9. ^{1}H NMR (600 MHz, methanol-d_4) and ^{13}C NMR (150 MHz, methanol-d_4) of DON-13-GSH

Figure S10. Composite omit maps of ligands DON-13-GSH (A1H25) and DON-13-cysteine (A1H24)

Figure S11. Formation of DON-10-GSH and DON-13-GSH by active site mutants of TaGST-02 and TaGST-10

Table S2. Students t-test of enzymatic DON-10-GSH synthesis compared to the negative control related to the results shown in Fig. 3. Displayed are the *p*-values of a two-tailed two-sample (equal variance) t-test (n = 3).

Time (h)	TaGST02	TaGST06	TaGST08	TaGST10	TaGST12
2	$4.0\ 10^{-08}$	$5.4\ 10^{-06}$	$1.1\ 10^{-05}$	$1.8\ 10^{-05}$	$5.4\ 10^{-07}$
6	$2.2\ 10^{-06}$	$1.3\ 10^{-04}$	$9.3\ 10^{-03}$	$2.3\ 10^{-05}$	$1.1\ 10^{-07}$
11	$5.8\ 10^{-07}$	$1.7\ 10^{-05}$	$7.5\ 10^{-04}$	$4.4\ 10^{-05}$	$7.4\ 10^{-07}$
24	$4.5\ 10^{-06}$	$4.6\ 10^{-06}$	$1.2\ 10^{-04}$	$1.9\ 10^{-02}$	$4.1\ 10^{-07}$

Table S3. Interface analysis summary of the TaGST-10 dimer (PDB code 9S3A) with PDBePISA (https://www.ebi.ac.uk/msd-srv/prot_int/cgi-bin/piserver, reference 74).

Selection range	Chain A		Chain B	
Symmetry operation	x,y,z		x,y,z	
Symmetry ID	0_555		1_555	
Number of atoms				
Interface	100	6.3%	98	6.3%
Surface	950	59.9%	927	60.0%
Total	1587	100.0%	1544	100.0%
Number of residues				
Interface	25	11.8%	24	11.6%
Surface	193	91.0%	187	90.3%
Total	212	100.0%	207	100.0%
Solvent-accessible area, Å²				
Interface	975.4	8.9%	974.6	9.3%
Total	10989.9	100.0%	10435.9	100.0%
Solvation energy, kcal/mol				
Isolated structure	-209.7	100.0%	-206.5	100.0%
Gain on complex formation	-7.9	3.8%	-8.1	3.9%
Average gain	-4.6	2.2%	-3.6	1.7%
p-Value	0.125		0.056	

Table S4. Hydrogen bonds and salt bridges involved in TaGST-10 dimerization. Analysis performed with PDBePISA (74).

**Hydrogen bonds**		Distance (Å)
A:PRO 67[O]	B:HIS 95[NE2]	3.62
A:GLU 80[OE2]	B:ARG 97[NH1]	3.26
A:GLU 80[OE2]	B:ARG 101[NH1]	3.16
A:GLU 80[OE2]	B:ARG 101[NH2]	3.49
A:HIS 95[NE2]	B:PRO 67[O]	3.38
A:ARG 97[NH1]	B:GLU 80[OE2]	3.62
A:ARG 101[NH1]	B:GLU 80[OE2]	2.94

**Salt bridges**		Distance (Å)
A:GLU 80[OE1]	B:ARG 101[NH1]	3.46
A:GLU 80[OE2]	B:ARG 97[NH1]	3.26
A:GLU 80[OE2]	B:ARG 101[NH1]	3.16
A:GLU 80[OE2]	B:ARG 101[NH2]	3.49
A:ARG 101[NH1]	B:GLU 80[OE1]	3.72
A:ARG 97[NH1]	B:GLU 80[OE2]	3.62
A:ARG 101[NH2]	B:GLU 80[OE2]	3.40
A:ARG 101[NH1]	B:GLU 80[OE2]	2.94

Table S5. Oligonucleotide primers for amplification of full-length wheat GST genes from genomic wheat DNA (cv. Chinese Spring).

ID	Name	Oligonucleotide sequence (5‘ –> 3‘)
4122	TaGST-01_5'UTR_fw	CAATTGCTACGTTCAAATGCATG
4123	TaGST-01_3'UTR_rv	TCCCTCCAACATAAGTTATTTAAC
4126	TaGST-02_upstr_fw	CCGGACAAAATGGGGTCG
4127	TaGST-02_3'UTR_rv	TTATTCAATGGAAGTCACGTC
4128	TaGST-03_fw+NdeI	agaacatATGGCGCCGGTGAAGGTG
4129	TaGST-03_3'UTR_rv+EcoRI	tgtgaattcGTAGCTATATACTTATAAGCAG
4130	TaGST-04_fw+NdeI	ttccgcatATGTCTCCGGTGAAGGTGT
4131	TaGST-04_3'UTR_rv	CAAGATTGATGGATGCATGG
4124	TaGST-06_3'UTR_rv	AAGAAGACGCGTTGACATACAC
4125	TaGST-06_5'UTR_fw	CACAAGTCGATTGACCCAACAC
4134	TaGST-07_fw+NdeI	agaacatATGGGGACGGAGGCGAAA
4135	TaGST-07_3'UTR_rv	TTGTTCGGCTAAAGTTTTCTC
4136	TaGST-08_fw+NdeI	tcagcatATGACGCATCGATCTTTTATAT
4137	TaGST-08_3'UTR_rv+EcoRI	atccgaattCAAGTTTTCAAACTGATAATACC
4510	TaGST-09_5'UTR_fw	CCACTTCATATAATAGACATACCACA
4511	TaGST-09_3'UTR_rv	CCAGCAGGTTACTTATTCTGG
4512	TaGST-10_upstr_fw	ACTCTTCAGTTTAAATATGCGATTAA
4513	TaGST-10_down_rv	CGTGAGCGTAGTAAGACGA
4514	TaGST-11_upstr_fw	CCATACAGGCCTAAGTGGAG
4515	TaGST-11_down_rv	GCAAAGACCGGGTTTTAAAC
4516	TaGST-12_5'UTR_fw	CCTTACACACACAGATCTAGATG
4517	TaGST-12_3'UTR_rv	CAAGAACAGAAATACGGATTTCC
4518	TaGST-15_upstr_fw	AGCTTCACATCTGCGATTAG
4519	TaGST-15_3'UTR_rv	CTGTATATACTTGCTAACTGACATG

Table S6. Oligonucleotide primers for amplification of wheat GST gene exons, assembly and cloning into expression vector pCA02 (58). Exons were assembled by overlap-extension PCR (OE-PCR) or with the NEBuilder HiFi DNA Assembly Master Mix (New England Biolabs).

ID	Name	Oligonucleotide sequence (5‘ –> 3‘)	Cloning method	Plasmid
4149	TaGST-01_fw+NdeI	tatcatATGGCAGCTCAAGGAGACC	Assembly of exons 1 and 2 by OE-PCR	pSI720
4150	TaGST-01-Exon1_rv+OL	ccgacataagcggagaatagTTTGTCGTCGACGTAGGCG	NdeI/EcoRI ligated to pCA02	
4151	TaGST-01-Exon2_fw	CTATTCTCCGCTTATGTCGG		
4152	TaGST-01_rv+EcoRI	tatgaattcTCACTTGGCACTCGCTGC		
4153	TaGST-02_GA-E1-fw	aatctctacttccaaggccatATGGCCGGAGGAGATGAC	Assembly of exons 1 and 2 by OE-PCR	pSI723
4154	TaGST-02_GA-E1-rv	gggcgacgagCTTATCGTCAACGTAGGCG	NdeI/EcoRI ligated to pCA02	
4155	TaGST-02_GA-E2-fw	tgacgataagCTCGTCGCCCCATGGGTA		
4156	TaGST-02_GA-E2-rv	aagcttgtcgacggagctcgaattcTTACTTGGTCTCTGAAGCAGCG		
4506	TaGST-03_GA-E1-fw	TCTCTACTTCCAAGGCCATATGGCGCCGGTGAAGGTGTT	Exon 2 was added as overhang to PCR fragment	pSI810
4507	TaGST-03_GA-E1_rv	GCGGGGATTTGCCCGAACGGGTTTCTGGCAAGGTGGTCGG	of exon 3	
4508	TaGST-03_GA-E2+3-fw	CCGTTCGGGCAAATCCCCGCGTTCCAGGACGGTGACCTCGTTCTCTTCGAGTCACGAGCGGTCGCAAAG	NEBuilder Assembly with NdeI/EcoRI digested	
4509	TaGST-03_GA-E3-rv	TTGTCGACGGAGCTCGAATTCTCATGGTGTCATGGTTGCACTGA	pCA02	
4157	TaGST-04_GA-E1-fw	gtgagaatctctacttccaaggccatATGTCTCCGGTGAAGGTG	NEBuilder assembly with NdeI/EcoRI digested	pSI742
4158	TaGST-04_GA-E1+2_rv	cgaacaggacgagatccccatcttggaacccaggcatcttcgcaaacggGTTTAACTGGACGTGTTGGG	pCA02	
4159	TaGST-04_GA-E3-fw	caagatggggatctcgtcctgttcgAGTCGCGCGCCATCGCCA	Exon 2 was added as overhang to PCR fragment	
4160	TaGST-04_GA-E3-rv	gccgcaagcttgtcgacggagctcgaattCTAGTACTGCGCACCTAGCTTAAACTTGGTAGG	of exon 1	
4161	TaGST-06-fw+NdeI	cagcatATGGCGGGCGAGAAGGG	No intron, direct NdeI/EcoRI ligation to pCA02	pSI770
4162	TaGST-06-rv+EcoRI	tgtgaattcTTACTCGACGCCGTGCATC		
4163	TaGST-07_GA-E1-fw	gtgagaatctctacttccaaggccatATGGGGACGGAGGCGAAA	Exon 2 was added as overhang to exons 1 and 3,	pSI731
4164	TaGST-07_GA-E1+2_rv	TCACCGTCCTGGAAAGCCGGGACCTGGCCAAAGAGGGTGCGGGCGAGATGCG	NEBuilder assembly with NdeI/EcoRI digested	
4165	TaGST-07_GA-E2+3-fw	CCCGGCTTTCCAGGACGGTGATCTCATCCTTTTCGAGTCGCGCGCGATTTCGA	pCA02	
4166	TaGST-07_GA-E3-rv	gccgcaagcttgtcgacggagctcgaattcTCATGACGCATCCATAAGCT		
4136	TaGST-08_fw+NdeI	tcagcatATGACGCATCGATCTTTTATAT	No intron, NdeI/EcoRI ligated to pCA02	pSI718
4167	TaGST-08_rv+EcoRI	tgtgaattcTTATTGGGTGCTTGCAGCAG		
4532	TaGST-09_GA-E1-fw	tctctacttccaaggccatATGTCGTCGGGGAAGCAGG	NEBuilder assembly with NdeI/EcoRI digested	pSI812
4533	TaGST-09_GA-E1_rv	CCGTAGGAAACTTGTCGTCGACGTACTGGGCC	pCA02	
4534	TaGST-09_GA-E2-fw	CGACGACAAGTTTCCTACGGCGATCCGG		
4535	TaGST-09_GA-E2-rv	ttgtcgacggagctcgaattcTCACTTGGAATTAGCACTAGCC		
4537	TaGST-10_GA-E1-fw	tctctacttccaaggccatATGGCCGGAGGAGGAAG	OE-PCR, NdeI/EcoRI ligated to pCA02	pSI828
4538	TaGST-10_GA-E1_rv	GGACGACGCTTTCACGAGCGTGCCGTCGATGTAAGC		
4539	TaGST-10_GA-E2-fw	CGCTCGTGAAAGCGTCGTCC		
4540	TaGST-10_GA-E2-rv	ttgtcgacggagctcgaattcTTAGTTTGTAGCGGCGGC		
4541	TaGST-11_GA-E1_fw	aatctctacttccaaggccatATGGCCGGAGCAGCAAA	NEBuilder assembly with NdeI/EcoRI digested	pSI815
4542	TaGST-11_GA-E1_rv	CCTTCACGAGCGTGTCCTCGATGAAGGC	pCA02	
4543	TaGST-11_GA-E2_fw	CGAGGACACGCTCGTGAAGGCGATGAACC		
4544	TaGST-11_GA-E2_rv	aagcttgtcgacggagctcgaattcTTAGTTTGCCGCAACAGCA		
4153	TaGST-02_GA-E1-fw	aatctctacttccaaggccatATGGCCGGAGGAGATGAC	NEBuilder assembly with NdeI/EcoRI digested	pSI818
4545	TaGST-12_GA-E1_rv	GGGTGACGAGCTTATCGTCAATGTAGGCGG	pCA02	
4546	TaGST-12_GA-E2_fw	TGACGATAAGCTCGTCACCCCATGGGTA		
4547	TaGST-12_GA-E2_rv	aagcttgtcgacggagctcgaattcTACTTGGTCTCCGAAGCAGC		
4548	TaGST-15_GA-E1_fw	aatctctacttccaaggccatATGACGCACGGAGGTATACA	NEBuilder assembly with NdeI/EcoRI digested	pSI821
4549	TaGST-15_GA-E1_rv	CCGGCAAGAGCTTGCTGTCGGCGTAGGC	pCA02	
4550	TaGST-15_GA-E2_fw	CGACAGCAAGCTCTTGCCGGCGTGGGT		
4551	TaGST-15_GA-E2_rv	aagcttgtcgacggagctcgaattcTCACGACAGCAGCTGGG		

Table S7. Oligonucleotide primers for creating TaGST-02, TaGST-10 and TaGST-12 constructs for expression as *N*-His_6-SUMO-tagged proteins in pET21a. The SUMO gene was amplified from yeast strain YPH499 DNA with primer pair 5249/5250, reamplified with 5260/5250 and further with 5263/5259 to introduce overhangs (NdeI-His_6 at 5´ and XhoI at 3´). The amplified fragment was ligated to pET21a using the NdeI/XhoI restriction sites, yielding pHM73. TaGST-02 and TaGST-12 genes were amplified with primer pairs 5686/5687 (template pSI723) and 5688/5689 (template pSI818), respectively. The resulting fragments were fused to XhoI digested pHM73 with the NEBuilder® HiFi DNA Assembly Master Mix (New England Biolabs). SUMO protease ULP1 was amplified from YPH499 DNA with oligos 5251/5252, reamplified with 5254/5255 and ligated to pET21a using NdeI/XhoI sites.

ID	Name	Oligonucleotide sequence (5‘ –> 3‘)
5249	SMT3 Fw1	ATGTCGGACTCAGAAGTCAATCAAGAAGCTAAGC
5250	SMT3 Rv1	CGTAGCACCACCAATCTGTTCTCTGTGAGC
5259	SUMO Rv Xho	TTTTCTCGAGACCACCAATCTGTTCTCTGTGAGCC
5260	SUMO Fw His6	TATACATATGCATCATCATCATCATCATGGCAGCCTGCAGTCGGACTCAG
5263	pET_SUMO_His_F2	CTTTAAGAAGGAGATATACATATGGCAAGCCATCATCATCATCATCATGGCAGCC
5251	ULP1 Fw1	CTTGTTCCTGAATTAAATGAAAAAGACGATGACC
5252	ULP1 Rv1	CTATTTTAAAGCGTCGGTTAAAATCAAATGGGC
5254	ULP1 Fw2 Nde	AAAcatatgCTTGTTCCTGAATTAAATGAAAAAGACG
5255	ULP1 Rv2 Xho	TTTTctcgagCTATTTTTCAAACTGCGGATGGCTCCAaagcttATGATGATGATGATGATGggatccTTTTAAAGCGTCGGTTAAAATCAAATGG
5686	TaGST02_SUMF	ACAGAGAACAGATTggtggtGCCGGAGGAGATGACTTGAAGCTGC
5687	TaGST02_SUMR	CAGTGGTGGTGGTGGTGGTGCTCGAGTTACTTGGTCTCTGAAGCAGCGGCG
5688	TaGST12_SUMF	ACAGAGAACAGATTggtggtGCCGGAGGAGATGACTTGAAGCTGC
5689	TaGST12_SUMR	CAGTGGTGGTGGTGGTGGTGCTCGAGCTACTTGGTCTCCGAAGCAGCGGC
5822	TaGST10_SUMF	ACAGAGAACAGATTggtggtGCCGGAGGAGGAAGCGAC
5823	TaGST10_SUMR	cagtggtggtggtggtggtgctcgagTTAGTTTGTAGCGGCGGCTGCG

Table S8. Oligonucleotide primers for site directed mutagenesis of TaGST02 and TaGST10

ID	Name	Oligonucleotide sequence (5‘ –> 3‘)
5820	C02V114S Fw	CTCGTCGCCCCATGGTCACAGTCGTTGAGGGC
5821	C02V114S Rv	CCATGGGGCGACGAGCTTATCGTC
5824	C10S116V Fw	CTCGTGAAAGCGTCGGTCCAGGCGTCCATGGC
5825	C10S116V Rv	CGACGCTTTCACGAGCGTGCCG
5826	C10V112S Fw	TCGACGGCACGCTCTCGAAAGCGTCGTCCC
5827	C10V112S Rv	GAGCGTGCCGTCGATGTAAGCG
5828	C10F220Y Fw	GGGCAAGCTGGTCGAGTATGCCATGGC
5829	C10F220Y Rv	CTCGACCAGCTTGCCCACGTCC

Table S9. Multiple reaction monitoring transitions of DON-10-GSH and DON-13-GSH

Analyte	Quantifier/Qualifier		Q1	Q3	Declustering potential (DP)	Collision energy (CE)	Cell exit potential (CXP)
		ESI mode	ion (m/z)	ion (m/z)	Energy (V)	Energy (V)	Energy (V)
DON-13-GSH.1	Quantifier	Positive	604.104	445.000	156	31	24
DON-13-GSH.2	Qualifier	Positive	604.104	262.900	156	39	14
DON-10-GSH.1	Quantifier	Negative	602.300	306.100	-55	-34	-21
DON-10-GSH.2	Qualifier	Negative	602.300	271.700	-70	-40	-14

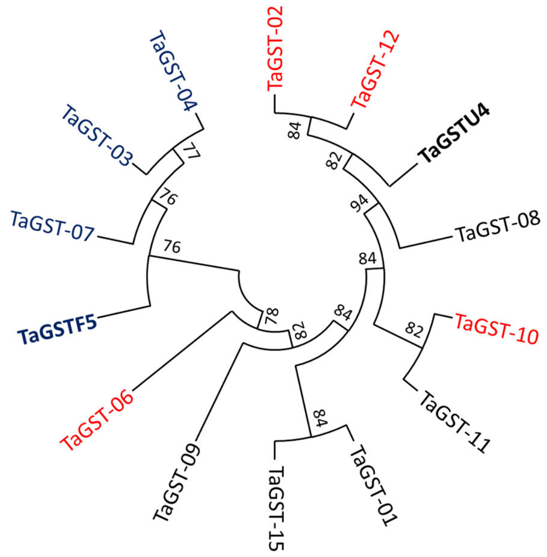

Figure S1. Neighbor-joining tree (with bootstrap values in %) of wheat GSTs selected in this study with previously characterized wheat GSTs of classes tau (TaGSTU4, 35) and phi (TaGSTF5, 28, 79). Phi class GSTs are highlighted blue, tau class GSTs showing activity with DON in red. Sequence alignment (ClustalW algorithm) and tree were created with MEGA 10 (77).

```
FGENESH 2.6 Prediction of potential genes in Triticum genomic DNA
Seq name:  test sequence
Length of sequence: 2424
Number of predicted genes 2: in +chain 1, in -chain 1.
Number of predicted exons 3: in +chain 2, in -chain 1.
Positions of predicted genes and exons: Variant 1 from 1, Score:210.656177
```

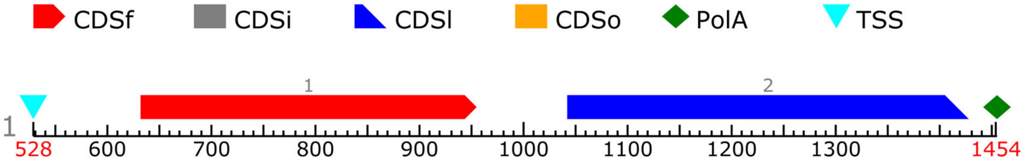


```
 1 +      TSS         528               -1.71
 1 +    1 CDSf        631 -       954   78.75       631 -       954     324
 1 +    2 CDSl       1041 -      1427   85.78      1041 -      1427     387
 1 +      PolA       1454                2.19
```

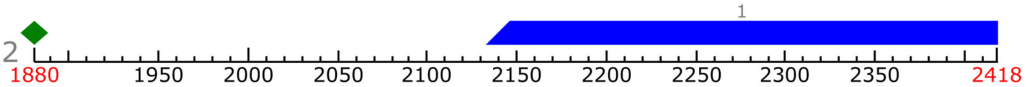


```
 2 -      PolA       1880                2.19
 2 -    1 CDSl       2132 -      2418   53.10      2132 -      2416     285
```

Predicted protein(s):
>FGENESH:[mRNA] 1 2 exon (s) 631 - 1427 711 bp, chain +
ATGGCCGGAGGAGATGACTTGAAGCTGCTGGGCGCTTGGGCAAGTCCATTTGTCACCAGGGTGAAGCTTGCGCTGAACTTCAAGGGC
CTGAGCTTCGAGGATGTCGAAGAGGACCTTAGCAACAAGAGCGAGCTCCTCCTCAGCTCGAACCCGGTGCACAAGAAGGTGCCCGTG
CTCGTCCACAACGGAAAACCCATTTGCGAGTCAGTGATCATCGTTCAGTACATCGATGAGGCGTTCGCCGGCATCGGCCCCGCTCTCCTT
CCCTCTGACCCCTACGAACGCGCCATTGCCCGTTTCTGGGCCGCCTACGTTGACGATAAGCTCGTCGCCCCATGGGTACAGTCGTTGAGG
GCCAAGACAGAGGAGGAGAAGTCCGAGGGGCTTAAGCAGACATTTGCCGCGGTGGAGACACTGGAAGGAGCCCTGCGGGAGTGCT
CCAAGGGAGAGGGCTACTTTGGTGGTGAGACCGTCGGGCTTGTGGACATTTCACTTGGGAGCCTGCTCTCCTGGTTGAACGCGACAG
AAGTGATGTCCGGAACCAAGATATTTGATCCTGTTAAGACTCCGCTCCTGGCAGCGTGGATGGAGCGCTTTAGCAAGCTCGATGCTGCC
AAGGCGGCGTTGCCAGAAGTTGATAGGGTGGTCGAATTTGCCAAGAAGAGACAAGCACAGGCTGCTGCCGCCGCCGCTGCTTCAGAG
ACCAAGTAA

>FGENESH: 1 2 exon (s) 631 - 1427 236 aa, chain +
MAGGDDLKLLGAWASPFVTRVKLALNFKGLSFEDVEEDLSNKSELLLSSNPVHKKVPVLVHNGKPICESVIIVQYIDEAFAGIGPALLPSDPYER
AIARFWAAYVDDKLVAPWVQSLRAKTEEEKSEGLKQTFAAVETLEGALRECSKGEGYFGGETVGLVDISLGSLLSWLNATEVMSGTKIFDPVK
TPLLAAWMERFSKLDAAKAALPEVDRVVEFAKKRQAQAAAAAAASETK

>FGENESH:[mRNA] 2 1 exon (s) 2132 - 2418 285 bp, chain -
GCCCTGAGGGAGTGCGCGAAGGGCAAGGGCTTCTTCGGCGGCGACAATGTCGGGCTCGTCGACGTCGTGCTAGGCAGCCTGCTCACG
TGGGTGCACGCGACCGAGGTGATGTCGGGGACCAAGATGTTTGACCCTTCTAAGACCCCACTGCTGGCCACGTGGATGGAGCGCTTCG
ACGAGCTTGCCGCTGCCAAGGCCGTCATGCCGGACGTTAACAGGATGGTCGAGTTCAAGACGAGGCAGGCGCAGGCCATCTCTGTCGC
TGCAGCTTCACAGCGTCAGTAA

>FGENESH: 2 1 exon (s) 2132 - 2418 94 aa, chain -
ALRECAKGKGFFGGDNVGLVDVVLGSLLTWVHATEVMSGTKMFDPSKTPLLATWMERFDE
LAAAKAVMPDVNRMVEFKTRQAQAISVAAASQRQ

Figure S2. FGENESH (http://www.softberry.com/) prediction of the TaGST-02 gene, IWGSC, INSDC wheat genome assembly GCA 900519105.1, Jul 2018; position 1D:262247455-262247781; 1D:262247865-262248248.

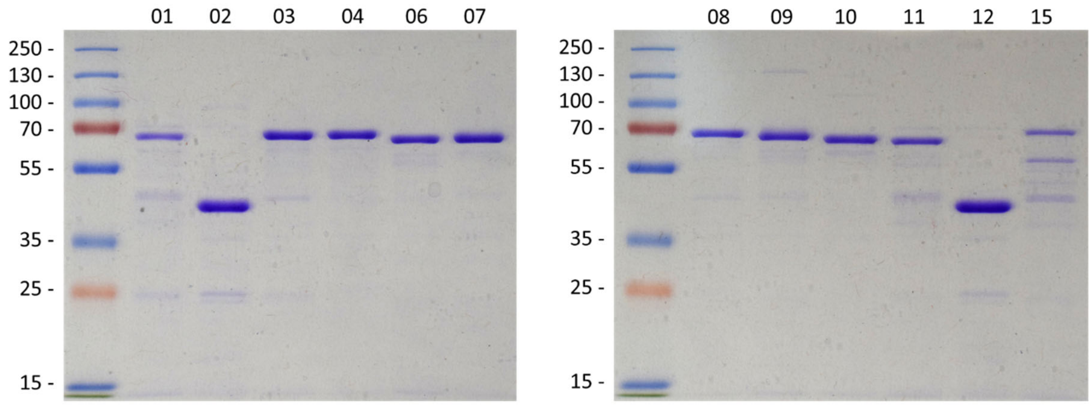

Figure S3. Sodium dodecyl sulfate polyacrylamide gel electrophoresis (SDS-PAGE) of one-step IMAC purified wheat GSTs with PageRuler Plus Prestained Protein Ladder (kDa, Thermo Scientific). All GSTs were expressed as *N*-His$_6$-MBP fusion proteins except for TaGST-02 and TaGST-12 (*N*-His$_6$-SUMO). The lane numbering corresponds to GST designation, calculated mass in kDa is given parentheses: TaGST-01 (69.8), TaGST-02 (38.0), TaGST-03 (68.2), TaGST-04 (69.5), TaGST-06 (68.7), TaGST-07 (67.7), TaGST-08 (72.9), TaGST-09 (69.4), TaGST-10 (68.4), TaGST-11 (68.3), TaGST-12 (38.0), TaGST-15 (73.0). 7.5 µg of total protein were loaded onto each lane.

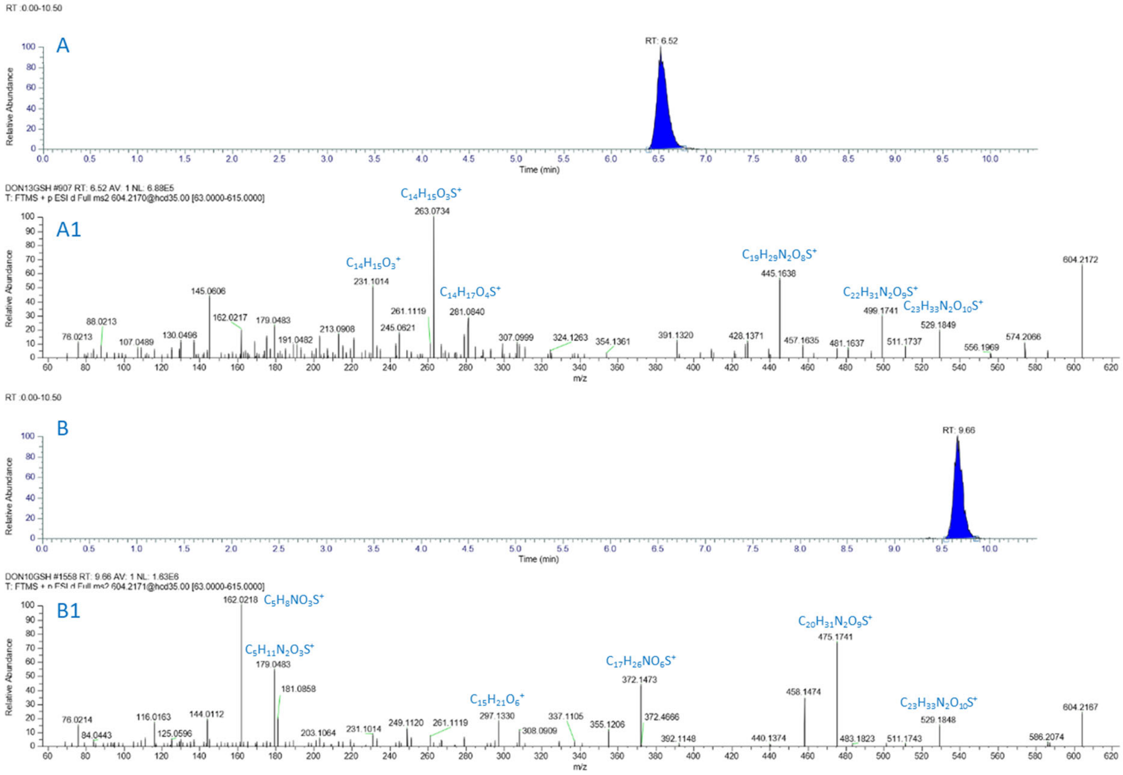

Figure S4. Extracted ion chromatogram of $[M+H]^+$ (*m/z* 604.2171 ± 5 ppm); (A) DON-13-GSH standard, parent ion [M+H]+, detected m/z 604.2172 (0.22ppm mass deviation), RT 6.52 min for full detected mass range (*m/z* 63–615); (B) DON-10-GSH standard, parent ion [M+H]+, detected m/z 604.2167 (-0.61ppm mass deviation), RT 9.66 min for full detected mass range (*m/z* 63–615). (A1) MS/MS fragmentation spectra of $[M+H]^+$ of DON-13-GSH standard; (B1) MS/MS fragmentation spectra of $[M+H]^+$ of DON-10-GSH standard.

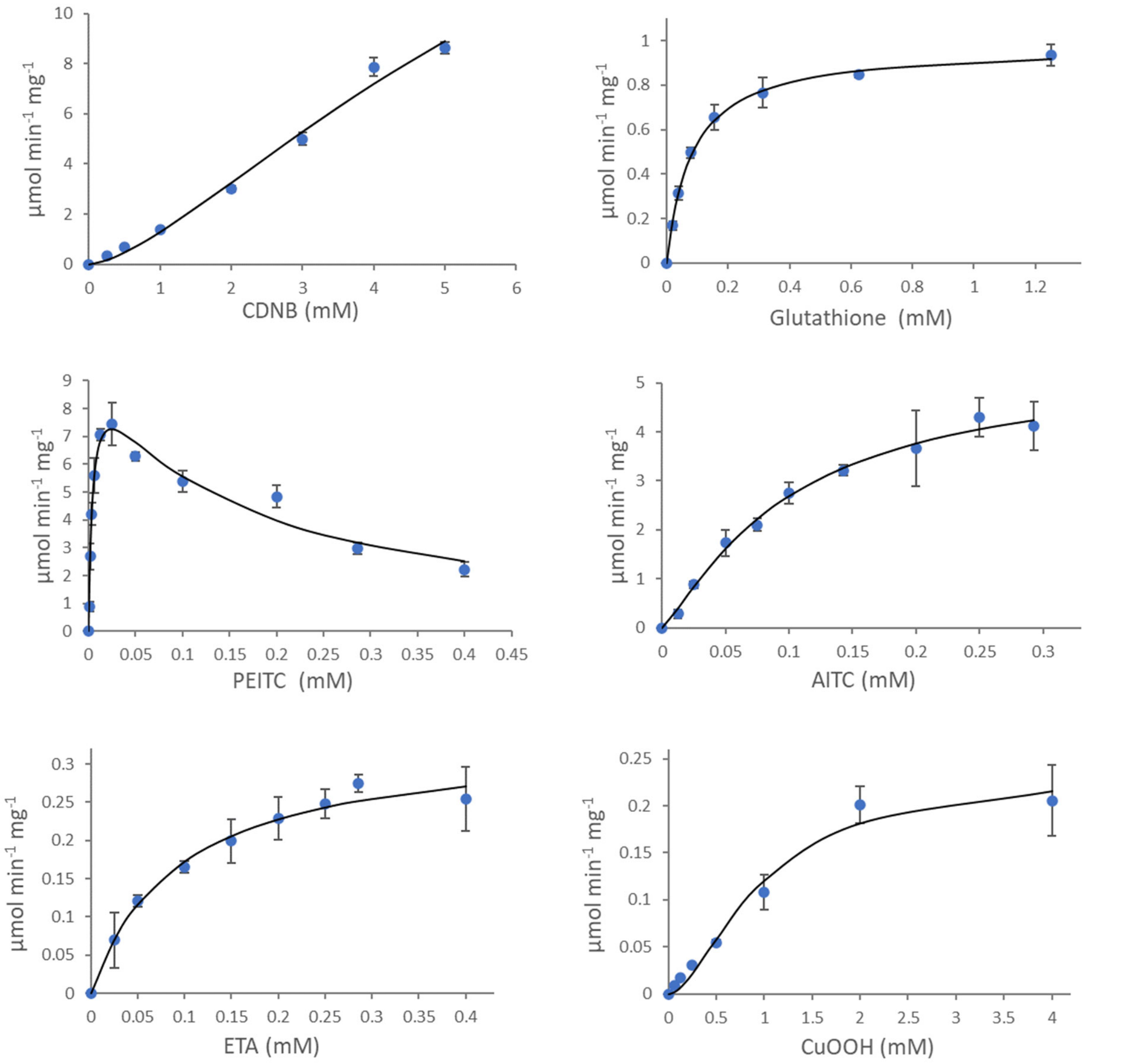

Figure S5. Steady state kinetic analysis of TaGST-02 with glutathione (GSH), 1-chloro-2,4-dinitrobenzene (CDNB), phenylethyl-isothiocyanate (PEITC), allyl-isothiocyanate (AITC), ethacrynic acid (ETA) and cumene hydroperoxide (CuOOH). The assays were carried out in 100 mM potassium phosphate pH 6.5, 20 °C, each measurement was performed in triplicate. The lines represent the curve fits using either the Hill (equation 1) or the Michaelis-Menten model (equation 2).

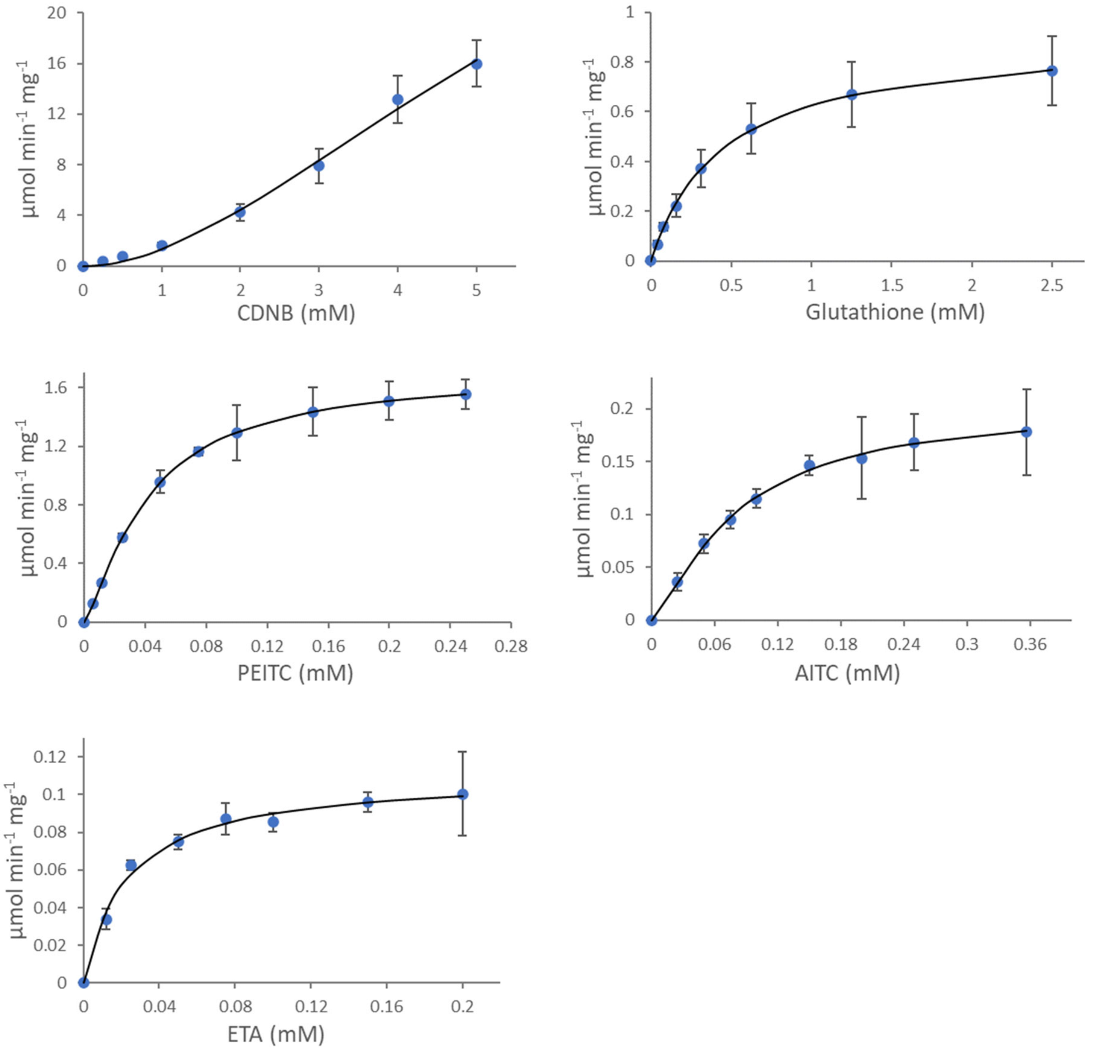

Figure S6. Steady state kinetic analysis of TaGST-10 with glutathione (GSH), 1-chloro-2,4-dinitrobenzene (CDNB), phenylethyl-isothiocyanate (PEITC), allyl-isothiocyanate (AITC) and ethacrynic acid (ETA). The assays were carried out in 100 mM potassium phosphate pH 6.5, 20 °C, each measurement was performed in triplicate. The lines represent the curve fits using either the Hill (equation 1) or the Michaelis-Menten model (equation 2).

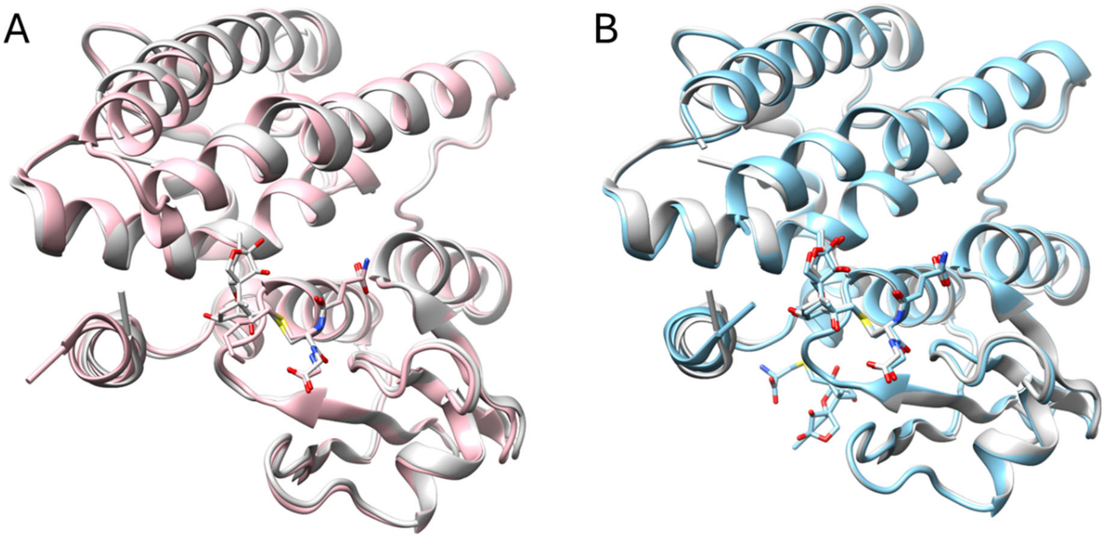

Figure S7. (**A**) Superposition of TaGST-10 (9S3A, chain B with DON-13-GSH, grey) on TaGSTU4 (1GWC, chain A binding *S*-hexyl-glutathione, pink) with RMSD 1.109 Å across all pairs. (**B**) Superposition of TaGST-10 chain A (grey) with chain B (light blue) with RMSD 0.592 Å across all pairs.

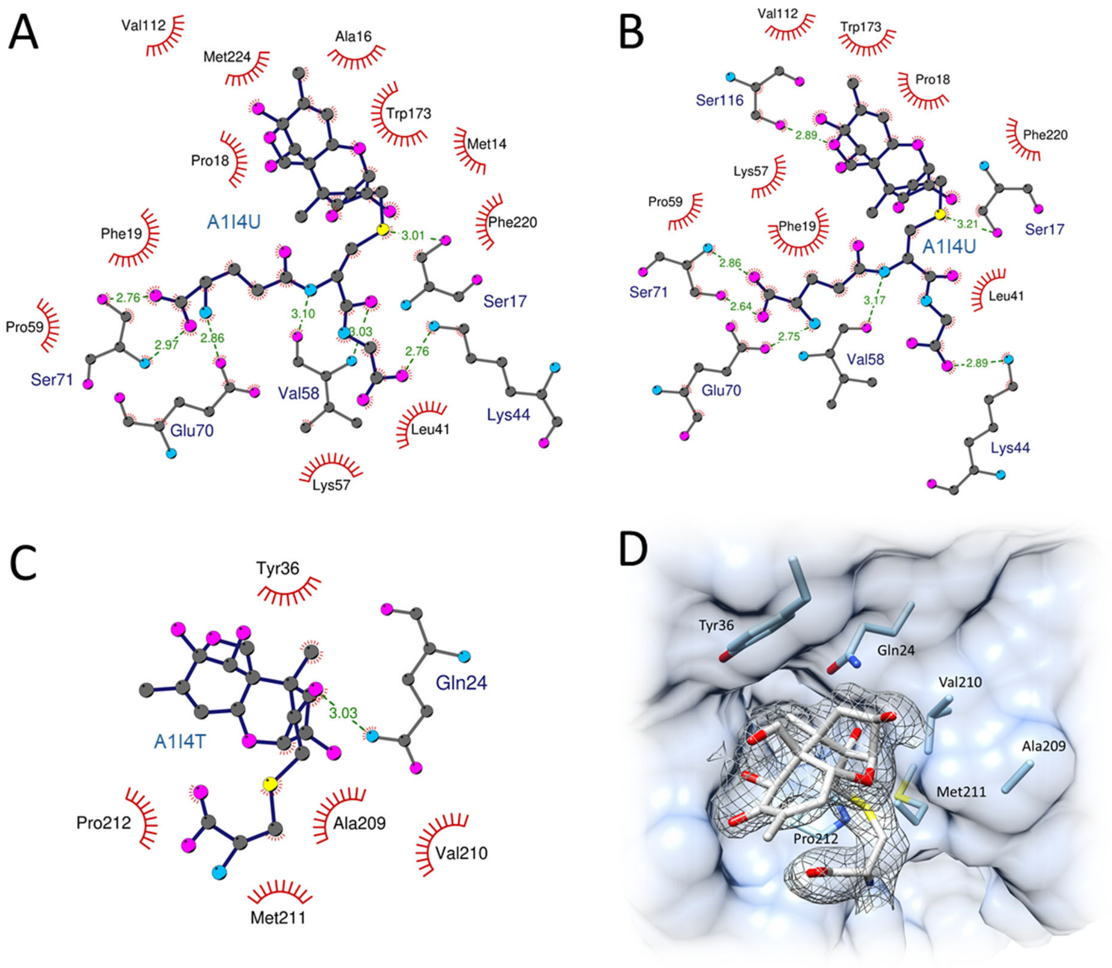

Figure S8. **A-C** LigPlot (75) representations of TaGST-10 (PDB ID 9S3A) active site residues interacting with ligands (**A**) DON-13-GSH (A1I4U) at chain A, (B) DON-13-GSH (A1I4U) at chain B, (**C**) DON-13-cysteine (A1I4T) at chain A. Hydrogen bonds are indicated with green dashed lines with distances in Å, non-bonded contacts are indicated by red spoked arcs. (**D**) DON-13-cysteine with electron density at contour level σ=1 bound to chain A of TaGST-10 (surface representation). Residues that make contact (max. distance 3.9 Å) with DON-13-cysteine are shown in stick representation.

A

Pos	¹H	¹³C
2	3.90 (d, 4.7)	81.6
3	4.59 (m)	71.0
4	2.03 (d, 13.8; d, 10.6)<br>1.45 (d, 13.8; d, 3.5)	46.0
5	-	52.0
6	-	52.4
7	3.97 (s)	78.0
8	-	105.9
9	-	143.5
10	5.40 (d, 4.0; q, 1.6)	123.3
11	4.86 (m)	77.9
12	-	82.8
13	3.54 (d, 13.1)<br>3.15 (d, 13.1)	37.2
14	1.33 (s)	16.2
15	4.21 (d, 8.5)<br>3.34 (d, 8.5)	70.6
16	1.78 (t, 1.6)	16.6
Glu-1	-	174.0
Glu-2	3.71 (t, 6.4)	55.4
Glu-3	2.15 (m)<br>2.10 (m)	27.9
Glu-4	2.58 (d, 15.6; d, 8.3; d, 6.4)<br>2.53 (d, 15.6; t, 6.4)	33.1
Glu-5	-	175.4
Cys-1	-	173.4
Cys-2	4.60 (d, 8.6; d, 5.0)	55.1
Cys-3	3.11 (d, 14.1; d, 5.0)<br>2.89 (d, 14.1; d, 8.7)	36.5
Gly-1	-	173.4
Gly-2	3.91 (d, 17.7)<br>3.89 (d, 17.6)	42.4

B

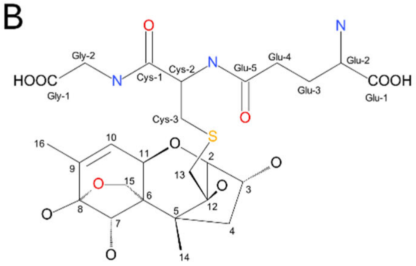

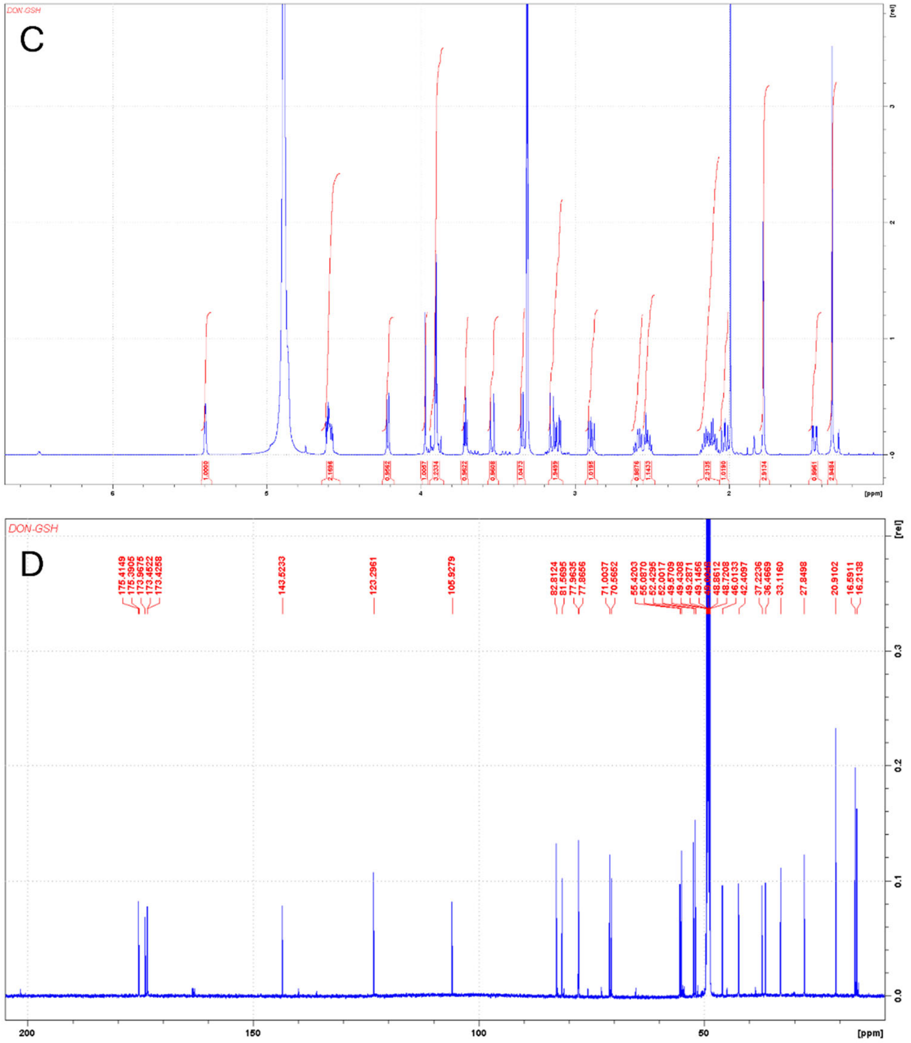

Figure S9. (**A**) ¹H NMR (δ, ppm; multiplicity; J, Hz) and ¹³C NMR data (δ, ppm) of DON-13-GSH (hemiketal form), (**B**) Structure of DON-13-GSH, (**C**) ¹H NMR (600 MHz, methanol-d_4) and (**D**) ¹³C NMR (150 MHz, methanol-d_4) of DON-13-GSH.

A

B

C

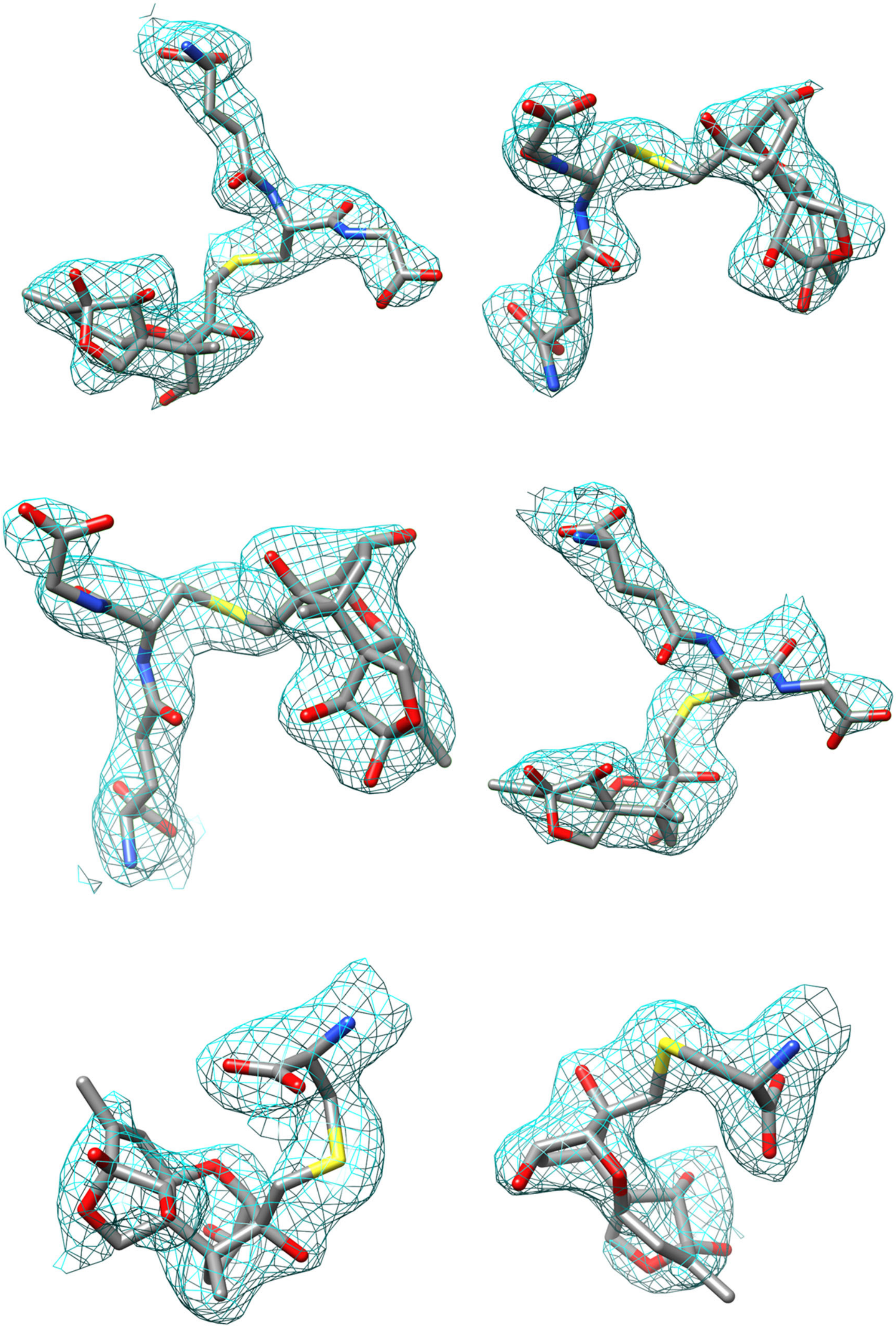

Figure S10. Composite omit maps of ligands modelled into the TaGST-10 structure (9S3A) with two displayed orientations each. (**A**) DON-13-GSH (A1I4U) at chain A, (**B**) DON-13-GSH (A1I4U) at chain B, (**C**) DON-13-cysteine (A1I4T) at chain A. Electron density is displayed at contour level σ=1.

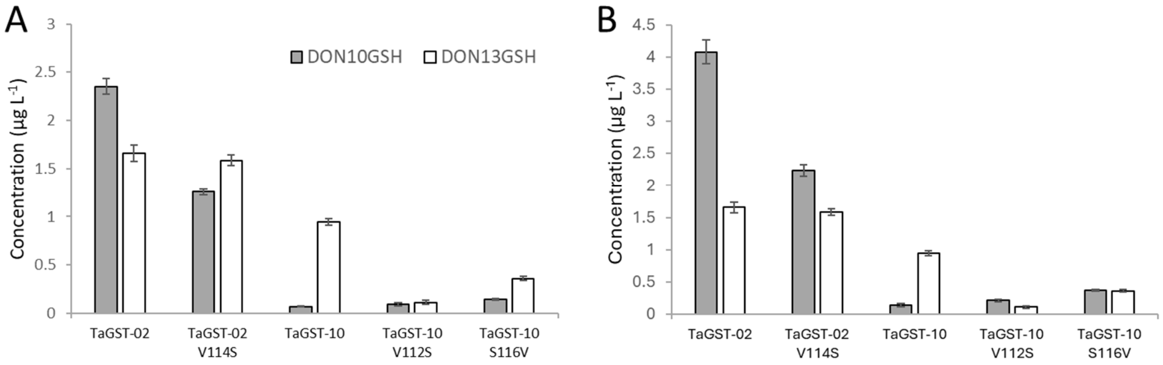

Figure S11. Formation of DON-10-GSH (Michael adduct) and DON-13-GSH (epoxide adduct) catalyzed by TaGST-02, TaGST-10 and several active site mutants created by site directed mutagenesis. 5 mg mL^{-1} of one-step IMAC-purified GSTs (His_6-SUMO-GST) were used in assays containing 30 mg L^{-1} (0.1 mM) DON, 5 mM GSH in 100 mM phosphate buffer, pH 6.5 at 20 °C. DON-10-GSH and DON-13-GSH were quantified by liquid chromatography coupled to tandem mass spectrometry (LC-MS/MS). Results are shown after 2 h (**A**) and 6 h (**B**) reaction time. The values displayed represent the average of triplicate determination, error bars indicate SD.
